# High expression levels of pyrimidine metabolic rate–limiting enzymes are adverse prognostic factors in lung adenocarcinoma: a study based on The Cancer Genome Atlas and Gene Expression Omnibus datasets

**DOI:** 10.1007/s11302-020-09711-4

**Published:** 2020-07-08

**Authors:** Haiwei Wang, Xinrui Wang, Liangpu Xu, Ji Zhang, Hua Cao

**Affiliations:** 1 Fujian Key Laboratory for Prenatal Diagnosis and Birth Defect, Fujian Maternity and Child Health Hospital,, Affiliated Hospital of Fujian Medical University, Fuzhou, Fujian China; 2 Key Laboratory of Technical Evaluation of Fertility Regulation for Non-human Primate,, National Health and Family Planning Commission, Fuzhou, Fujian China; 3 State Key Laboratory for Medical Genomics, Shanghai Institute of Hematology, Rui-Jin Hospital Affiliated to School of Medicine, Shanghai Jiao Tong University, Shanghai, China

**Keywords:** Lung adenocarcinoma, Pyrimidine metabolic rate–limiting enzymes, Purinergic receptors, Gene expression omnibus, The Cancer genome atlas

## Abstract

Reprogramming of metabolism is described in many types of cancer and is associated with the clinical outcomes. However, the prognostic significance of pyrimidine metabolism signaling pathway in lung adenocarcinoma (LUAD) is unclear. Using the Gene Expression Omnibus (GEO) and The Cancer Genome Atlas (TCGA) datasets, we found that the pyrimidine metabolism signaling pathway was significantly enriched in LUAD. Compared with normal lung tissues, the pyrimidine metabolic rate–limiting enzymes were highly expressed in lung tumor tissues. The high expression levels of pyrimidine metabolic–rate limiting enzymes were associated with unfavorable prognosis. However, purinergic receptors P2RX1, P2RX7, P2RY12, P2RY13, and P2RY14 were relatively downregulated in lung cancer tissues and were associated with favorable prognosis. Moreover, we found that hypo-DNA methylation, DNA amplification, and TP53 mutation were contributing to the high expression levels of pyrimidine metabolic rate–limiting enzymes in lung cancer cells. Furthermore, combined pyrimidine metabolic rate–limiting enzymes had significant prognostic effects in LUAD. Comprehensively, the pyrimidine metabolic rate–limiting enzymes were highly expressed in bladder cancer, breast cancer, colon cancer, liver cancer, and stomach cancer. And the high expression levels of pyrimidine metabolic rate–limiting enzymes were associated with unfavorable prognosis in liver cancer. Overall, our results suggested the mRNA levels of pyrimidine metabolic rate–limiting enzymes CAD, DTYMK, RRM1, RRM2, TK1, TYMS, UCK2, NR5C2, and TK2 were predictive of lung cancer as well as other cancers.

**Electronic supplementary material:**

The online version of this article (10.1007/s11302-020-09711-4) contains supplementary material, which is available to authorized users.

## Background

Lung cancer is one of the most commonly diagnosed cancer and the leading cause of cancer-related mortality [1–3]. Although some improvements of treatment have been achieved in the past few decades, the 5-year survival rate of lung cancer patients is still low [4, 5]. Lung cancer is a heterogeneous disease, including small cell lung cancer and non-small cell lung cancer (NSCLC) [6]. NSCLC accounts for the 85% of lung cancer cases and could be further divided into 3 major pathologic subtypes: lung adenocarcinoma (LUAD), lung squamous cell carcinoma (LUSC), and large-cell carcinoma [7]. Each subtype of NSCLC demonstrates different molecular profiles and different drug response [8, 9]. Although gene alterations [10, 11], mRNA expression signature [12, 13], microRNA profiles [14, 15], long non-coding RNAs [16, 17], immune signature [18], and tumor microenvironment [19] are used for the prognosis of NSCLC, more candidate biomarkers are needed.

Reprogramming of cell metabolism is a hallmark of cancer [20]. Cancer cells increase glucose uptake and utilize aerobic glycolysis to facilitate the uncontrolled cell proliferation [21]. Glycolysis-related gene signature is associated with the overall survival of LUAD patients [22]. Besides the misregulation of glucose metabolism, the pyrimidine metabolism is also disrupted during the development of cancer [23]. The disruption of the pyrimidine metabolism is reflected by the malfunctions of the pyrimidine metabolic rate–limiting enzymes. The high expression levels of pyrimidine metabolic rate–limiting enzymes CAD, CTPS, CTPS2, DHODH, DTYMK, NT5C2, NT5C3, RRM1, RRM2, TK1, TK2, TYMS, UCK2, and UCKL1 are illustrated in poorly differentiated liver cancer patients and correlated poor clinical outcomes [24]. Inhibition of pyrimidine synthesis by targeting pyrimidine metabolic rate–limiting enzymes DHODH and CAD could accentuate the molecular therapy response in glioblastoma [25]. Also, inhibition of pyrimidine synthesis sensitizes triple-negative breast cancer cells to chemotherapy [26]. However, the prognostic significance of the pyrimidine metabolism signaling pathway in LUAD is unclear.

In the present study, we used large cohorts of lung cancer patients derived from Gene Expression Omnibus (GEO) and The Cancer Genome Atlas (TCGA) datasets to demonstrate the prognostic significance of pyrimidine metabolic rate–limiting enzymes and purinergic receptors in LUAD. Overall, the analysis of GEO and TCGA datasets allowed an improved understanding of the functions of pyrimidine metabolic rate–limiting enzymes and purinergic receptors. The results also indicated the potential biomarkers of the pyrimidine metabolic rate–limiting enzymes for further clinical studies.

## Methods

### Data collection

The TCGA LUAD and LUSC gene expression, DNA mutation, and DNA methylation, along with the clinical datasets, were downloaded from the TCGA hub (https://tcga.xenahubs.net). The LUAD and LUSC gene expression data was generated from RNA-seq and the DNA methylation data was generated from Illumina HumanMethylation450 Bead Chip. Gene expression data derived from bladder urothelial carcinoma (BLCA), breast invasive carcinoma (BRCA), colon adenocarcinoma (COAD), esophageal carcinoma (ESCA), head and neck squamous cell carcinoma (HNSC), kidney renal clear cell carcinoma (KIRC), kidney renal papillary cell carcinoma (KIRP), liver hepatocellular carcinoma (LIHC), stomach adenocarcinoma (STAD), and thyroid cancer (THCA) were also downloaded from TCGA hub.

The gene expression series matrix of normal and cancerous lung tissues was downloaded from the GEO website (www.ncbi.nlm.nih.gov/geo) and included GSE7670, GSE10072, GSE18842, GSE19188, GSE27262, GSE30219, GSE31210, GSE31908, GSE33532, and GSE75324 datasets. The DNA methylation data of patients with LUAD was downloaded from the GEO datasets with GEO number GSE32867 and GSE62948. All the GEO expression datasets were based on Affymetrix Human Genome microarray.

**Fig. 1:**
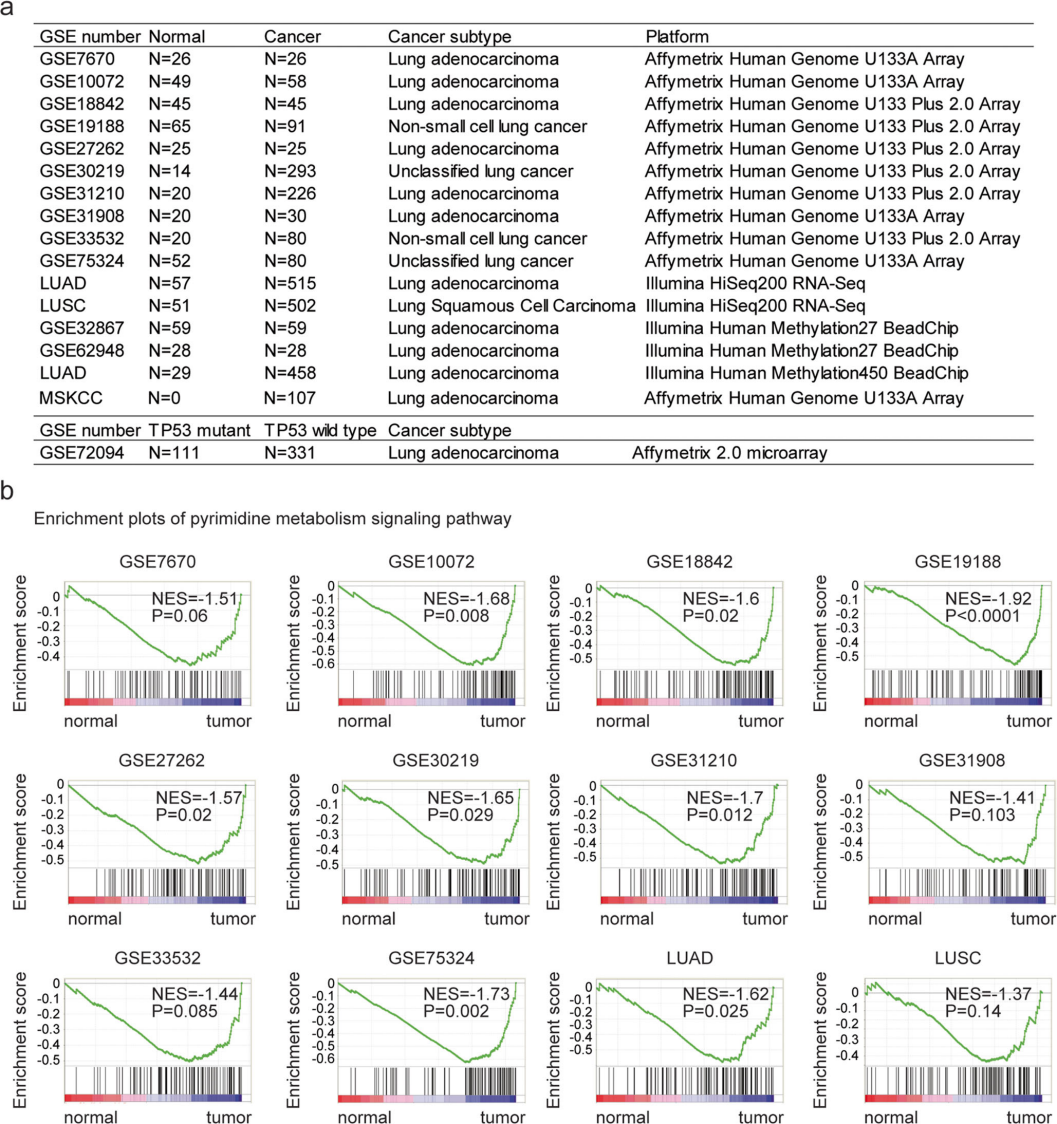
Pyrimidine metabolism signaling pathway is highly enriched in lung cancer. **a** Table showed the detailed GEO datasets and TCGA datasets used in this study. **b** Enrichment plots demonstrated the enriched pyrimidine metabolism signaling pathway in GSE7670, GSE10072, GSE18842, GSE19188, GSE27262, GSE30219, GSE31210, GSE31908, GSE33532, GSE75324, and TCGA LUAD, LUSC datasets. Enrichment of normalized enrichment score (NES) and *P* values were presented. LUAD: lung adenocarcinoma; LUSC: lung squamous cell carcinoma;

Clinical and raw data of MSKCC dataset are downloaded from http://cbio.mskcc.org/Public/lung_array_data/ [27]. The detailed description of the collected data used in this study is illustrated in Fig. 1a.

### Gene Expression Omnibus data processing

The GEO expression datasets were processed using R software (version 3.5.0, https://www.r-project.org/). The matrix file of each dataset was annotated with corresponding platform. When multiple probes corresponded to the same gene symbol, the expression values were averaged using “plyr” package (version 1.8.5) in R software. Plyr package includes multiple tools for splitting, applying, and combining data and could be downloaded from bioconductor (https://cran.r-project.org/web/packages/plyr/index.html). The different gene expression between normal and lung cancer samples was determined using paired Student’s *t* test. The different DNA methylation intensity between normal and lung cancer samples was also determined using paired Student’s *t* test.

### Gene set enrichment analysis

The metabolic singling pathways enriched in lung cancer gene expression profiling were determined using Gene set enrichment analysis (GSEA) software (version 2.0) [28]. The GSEA software and the signaling pathways gene sets were downloaded from the GSEA website (www.broad.mit.edu/gsea/index.html). Genes were ranked by the signal-to-noise ratio, and statistical significance was determined by 1000 gene set permutations. The results of significance should meet the criteria of nominal *P* value less than 0.05.

### Heatmap presentation

Heatmaps were created by R software “pheatmap” package (version 1.0.12). The pheatmap package and the basic usage were downloaded from bioconductor (https://cran.r-project.org/web/packages/pheatmap/). The clustering scale was determined by the “average” method. The clustering distance was determined by the “correlation” method. Other parameters were provided in the usage of the pheatmap.

### Survival analysis using Gene Expression Omnibus dataset

The Kaplan-Meier plotter (https://kmplot.com/analysis/) [29, 30] was used to identify the association between the expression levels of the pyrimidine metabolic rate–limiting enzymes, purinergic receptors, and overall survival in lung cancer derived from GEO datasets. The Kaplan-Meier plotter is an online survival analysis tool to rapidly assess the prognostic effects of genes using GEO microarray data. The patients were divided by the auto select best cutoff using the expression of the pyrimidine metabolic rate–limiting enzymes. *P* values were determined using Log-rank test.

### Survival analysis using The Cancer Genome Atlas dataset

R statistics software “survival” package (version 3.1–8) was used to identify the clinical influence of pyrimidine metabolic rate–limiting enzymes on overall survival in patients derived from TCGA LUAD, LUSC, LIHC, BRCA, and STAD datasets. The survival package and the basic usage were downloaded from bioconductor (https://cran.r-project.org/web/packages/survival/index.html). The patients were divided into two clusters based on the mean expression levels of genes. Kaplan-Meier estimator was applied to determine the clinical outcomes in patients with high expression levels and low expression levels of genes. *P* values were determined using Log-rank test.

### Oncoprints of the pyrimidine metabolic rate–limiting enzymes

The genomic alterations of pyrimidine metabolic rate–limiting enzymes in LUAD patients were downloaded from cbioportal (version 3.2.0) based on the TCGA datasets (http://www.cbioportal.org/index.do).

### Correlation plots of the pyrimidine metabolic rate–limiting enzymes

Correlation plots of the pyrimidine metabolic rate–limiting enzymes were created using the “corrplot” package (version 0.84) in R. The corrplot package and the basic usage were downloaded from bioconductor (https://cran.r-project.org/web/packages/corrplot/index.html). The Spearman’s correlation test was used to demonstrate the correlation efficiency.

### Multivariate Cox regression

Multivariate Cox regression was analyzed by “coxph” method in R software survival package (version 3.1–8). The survival package and the basic usage were downloaded from bioconductor (https://cran.r-project.org/web/packages/survival/index.html). Log-rank test was used to calculate the *P* values.

### Statistical analysis

The box plots were generated from GraphPad Prism software (version 5.0). Statistical analysis was performed using the paired Student’s *t* test using R software. *P* value less than 0.05 was chosen to be significantly different.

## Results

### Pyrimidine metabolism signaling pathway is highly enriched in lung tumor samples across different datasets

In order to reveal the metabolism-related transcriptional profiling in lung cancer, we analyzed lung cancer patients with expression data from previously published GEO datasets. Totally, 1290 samples were collected from ten previously published datasets based on Affymetrix gene microarray platforms, including 336 normal lung samples and 954 lung tumor samples. Most of the lung cancer patients belonged to LUAD subtype. A detailed description of the collected data used in this study is illustrated in Fig. 1a.

We then identified the enriched metabolic signaling pathways in patients with lung cancer using the GSEA assay. Among all the enriched metabolic signaling pathways, the pyrimidine metabolism signaling pathway was significantly enriched in seven out of ten datasets, including GSE10072, GSE18842, GSE19188, GSE27262, GSE30219, GSE31210, and GSE75324 datasets, representing the most frequently enriched metabolic signaling pathway (Fig. 1b). Only in GSE7670, GSE31908, and GSE33532 three datasets, the pyrimidine metabolism signaling pathway was not significantly correlated with the transcriptional profiling of lung cancer (Fig. 1b).

Using the TCGA lung cancer dataset, we found that the pyrimidine metabolism signaling pathway was positively associated with the transcriptional profiling of lung cancer in LUAD dataset (Fig. 1b). However, in another subtype of lung cancer LUSC, pyrimidine metabolism signaling pathway was not highly enriched (Fig. 1b).

### Pyrimidine metabolic rate–limiting enzymes are upregulated in lung cancer cells across different datasets

**Fig. 2:**
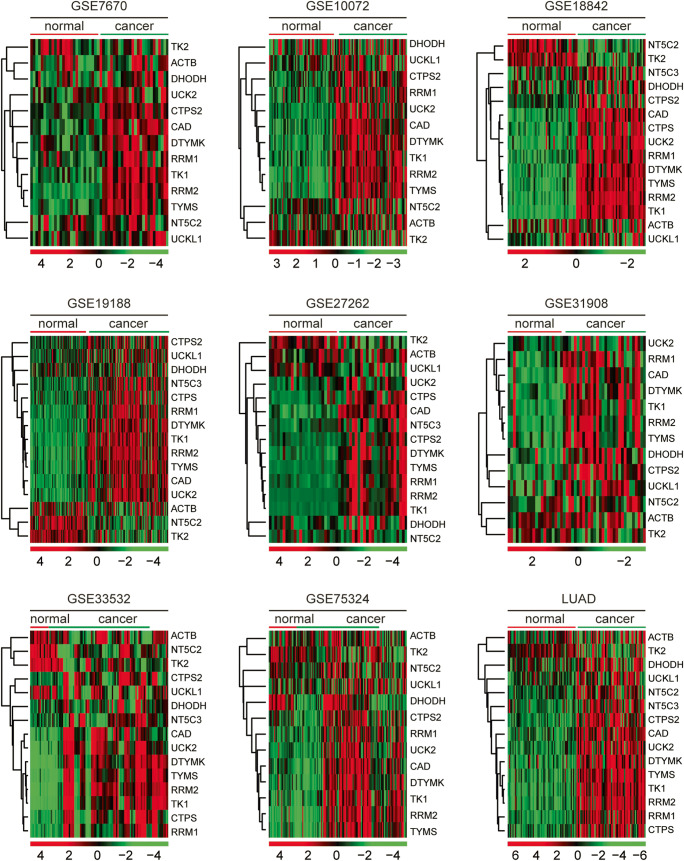
Pyrimidine metabolic rate limiting enzymes are up-regulated in lung cancer cells across different datasets. Heatmaps demonstrated the expression levels of pyrimidine metabolic rate–limiting enzymes in normal lung tissues and lung cancer tissues in GSE7670, GSE10072, GSE18842, GSE19188, GSE27262, GSE31908, GSE33532, GSE75324, and TCGA LUAD datasets. Upregulated (red), downregulated (green), and unchanged (black) genes were delineated. The expression levels of β-actin (ACTB) in normal and cancer tissues were used as control

The pyrimidine metabolism signaling pathway was involving multiple genes. Previous results suggested that pyrimidine metabolism was highly controlled by pyrimidine metabolic rate–limiting enzymes [24]. CAD, CTPS, CTPS2, DHODH, DTYMK, NT5C2, NT5C3, RRM1, RRM2, TK1, TK2, TYMS, UCK2, and UCKL1 were reported pyrimidine metabolic rate–limiting enzymes [24]. The expression levels of those pyrimidine metabolic rate–limiting enzymes in lung normal and tumor tissues were investigated in GSE7670, GSE10072, GSE18842, GSE19188, GSE27262, GSE31908, GSE33532, and GSE75324 datasets. As illustrated in the heatmaps, pyrimidine metabolic rate–limiting enzymes CAD, CTPS, CTPS2, DHODH, DTYMK, NT5C3, RRM1, RRM2, TK2, TYMS, UCK2, and UCKL1 were upregulated in lung cancer tissues (Fig. 2). However, TK2 and NT5C2 were relatively downregulated in lung cancer tissues (Fig. 2).

Similar results were derived from TCGA LUAD dataset. Compared with the normal lung tissues, pyrimidine metabolic rate–limiting enzymes CAD, CTPS, CTPS2, DTYMK, NT5C3, RRM1, RRM2, TK2, TYMS, UCK2, and UCKL1 were all highly expressed in lung cancer tissues. However, TK2 and NT5C2 were downregulated in lung cancer tissues (Fig. 2). And there was no significant difference of β-actin (ACTB) expression levels in normal and lung cancer tissues (Fig. 2).

### The expression levels of purinergic receptors in lung cancer cells

**Fig. 3:**
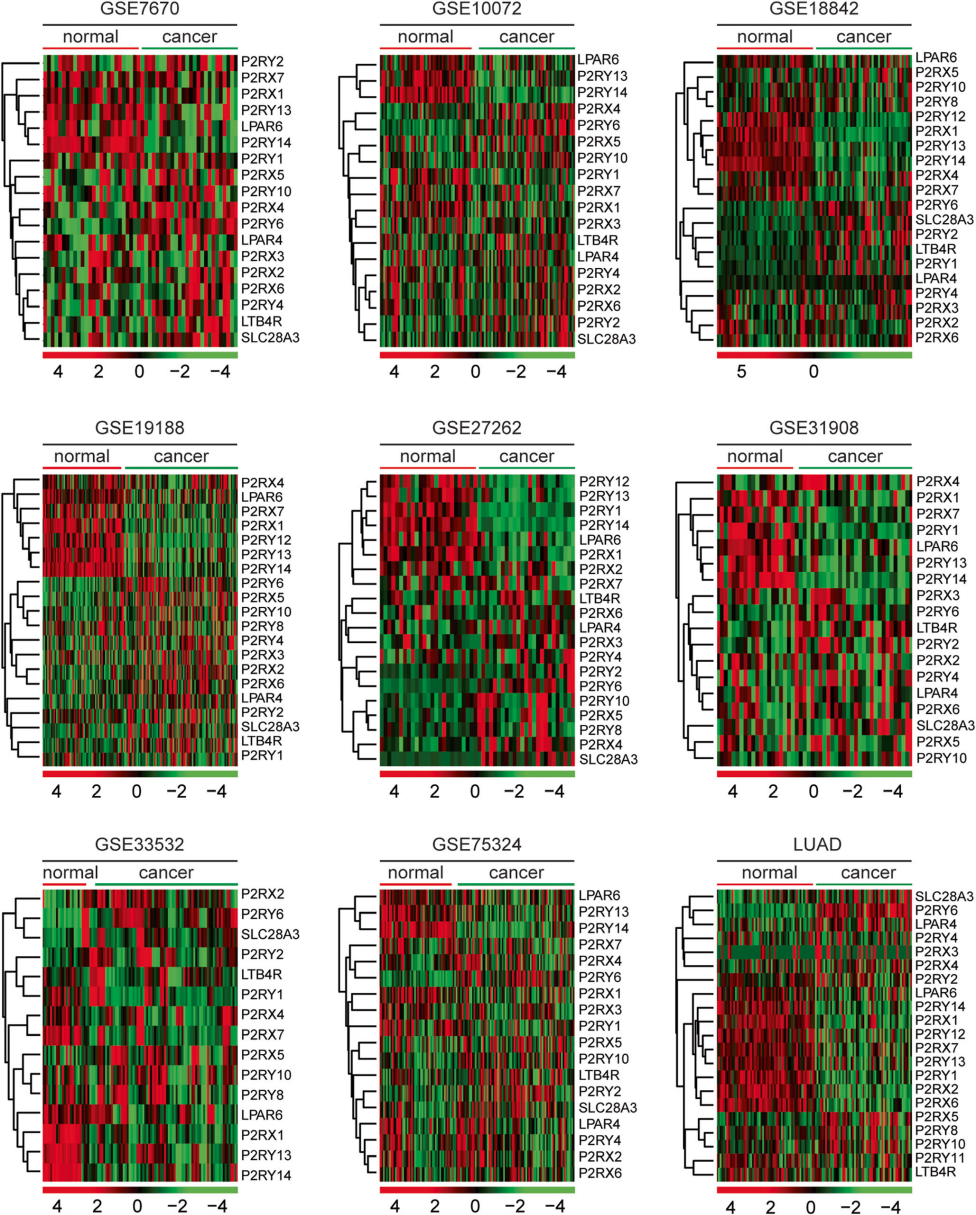
The expression levels of purinergic receptors in lung cancer cells. Heatmaps demonstrated the expression levels of purinergic receptors in normal lung tissues and lung cancer tissues in GSE7670, GSE10072, GSE18842, GSE19188, GSE27262, GSE31908, GSE33532, GSE75324, and TCGA LUAD datasets

Purinergic receptors comprise two different sub-families, ionotropic P2X and metabotropic P2Y receptors [31, 32]. Next, we determined the expression levels of P2X sub-families P2RX1–7 and P2Y receptors P2RY1, P2RY2, P2RY4, P2RY5 (LPAR6), P2RY6, P2RY7 (LTB4R), P2RY8, P2RY9 (LPAR4), P2RY10–14 in normal lung tissues and lung cancer tissues. As depicted in GSE7670, GSE10072, GSE18842, GSE19188, GSE27262, GSE31908, GSE33532, GSE75324, and TCGA LUAD datasets, compared with the normal lung tissues, purinergic receptors P2RX1, P2RX7, P2RY12, P2RY13, and P2RY14 were relatively downregulated in lung cancer tissues (Fig. 3). However, the expression levels of other purinergic receptors in normal and lung cancer tissues were not significantly different (Fig. 3). Also, the expression levels of nucleoside transporter SLC28A3 were not changed in lung cancer tissues (Fig. 3).

### Pyrimidine metabolic rate–limiting enzymes are upregulated in metastatic lung cancer cells and associated with lung cancer recurrence

**Fig. 4:**
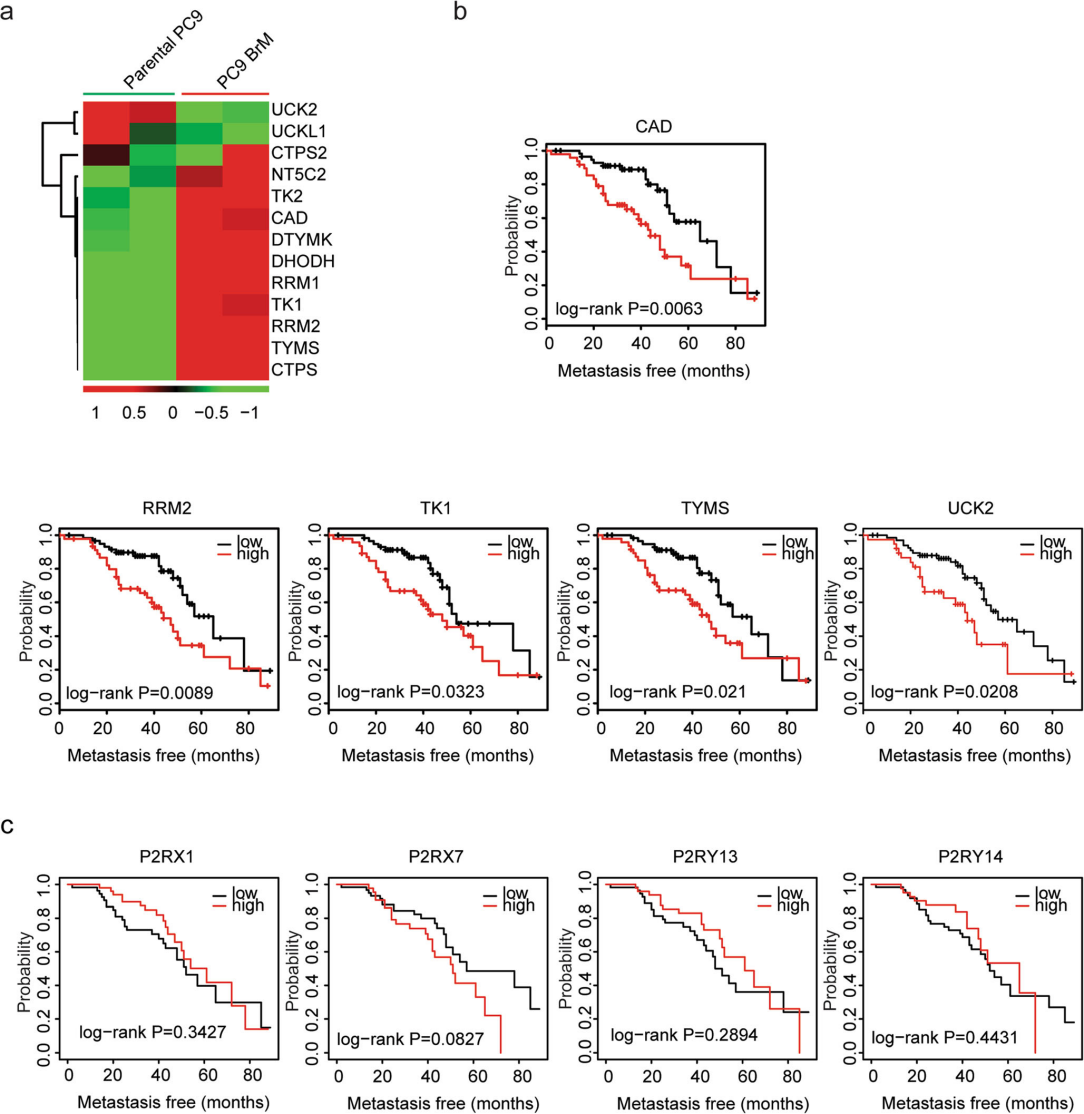
Pyrimidine metabolic rate–limiting enzymes are upregulated in metastatic lung cancer cells and associated with lung cancer recurrence. **a** Heatmaps demonstrated the expression levels of pyrimidine metabolic rate–limiting enzymes in parental PC9 cells and PC9 BrM cells. **b** The Kaplan-Meier plotters demonstrated the associations between pyrimidine metabolic rate–limiting enzymes and lung cancer recurrence using the MSKCC dataset. The log-rank test was used to determine the overall survival *P* value. **c** The Kaplan-Meier plotters demonstrated the associations between purinergic receptors P2RX1, P2RX2, P2RY13, and P2RY14 and lung cancer recurrence using the MSKCC dataset

LUAD cells can spread to the lymph nodes, adrenal glands, bones, and the brain [33]. PC9 BrM is a sub-population cells lines derived from parental PC9 LUAD cells, and with high brain metastasis [34]. We found that compared with parental PC9 cells, pyrimidine metabolic rate–limiting enzymes NT5C2, TK2, CAD, DTYMK, DHODH, RRM1, TK1, RRM2, TYMS, and CTPS were all highly expressed in PC9 BrM cells (Fig. 4a). Moreover, using MSKCC dataset, we showed that LUAD patients with high expression levels of CAD, RRM2, TK1, TYMS, or UCK2 were with high recurrence probability (Fig. 4b). However, purinergic receptors P2RX1, P2RX2, P2RY13, and P2RY14 were not associated with the tumor recurrence of lung cancer (Fig. 4c).

### Expression levels of pyrimidine metabolic rate–limiting enzymes are associated with the tumor overall survival in lung cancer: analysis from Gene Expression Omnibus datasets

**Fig. 5:**
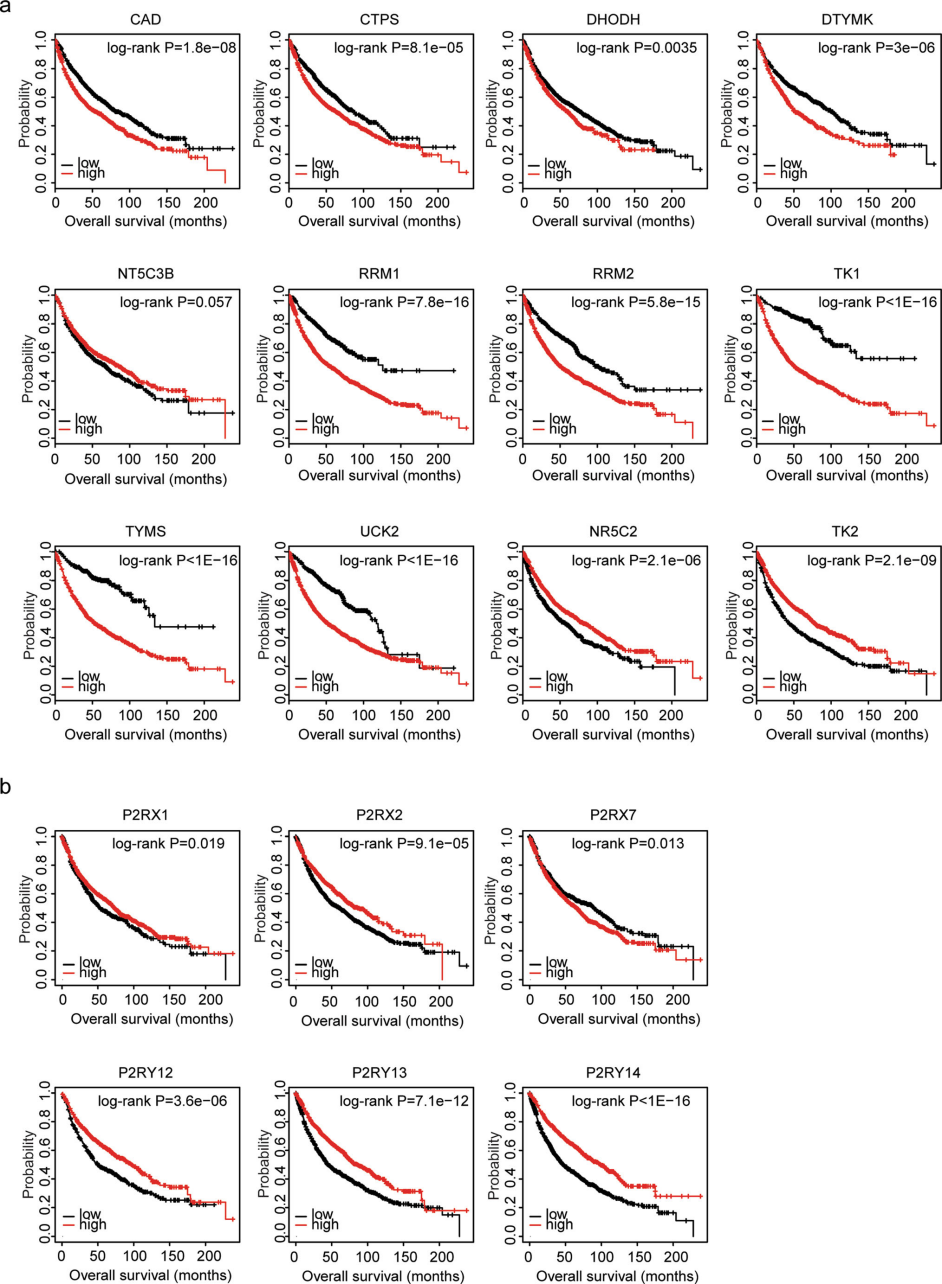
Expression levels of pyrimidine metabolic rate limiting enzymes are associated with the tumor overall survival in lung cancer: analysis from GEO datasets. **a** The Kaplan-Meier plotters demonstrated the prognostic effects of the expression levels of pyrimidine metabolic rate–limiting enzymes TK1, UCK2, CAD, RRM1, RRM2, DTYMK, TYMS, TK2, and NR5C2 in lung cancer using the integrated GEO datasets. The patients were divided into two groups by the auto select best cutoff based on the expression levels of the pyrimidine metabolic rate–limiting enzymes. The log-rank test was used to determine the overall survival *P* value. **b** The Kaplan-Meier plotters demonstrated the prognostic effects of the expression levels of purinergic receptors P2RX1, P2RX2, P2RX7, P2RY12, P2RY13, and P2RY14 in lung cancer

The Kaplan-Meier plotter is an online survival analysis tool to rapidly assess the prognostic effects of genes using the integrated GEO microarray data derived from 1926 lung cancer patients [29, 30]. Using Kaplan-Meier plotter, the present study showed that high expression levels of pyrimidine metabolic rate–limiting enzymes CAD, CTPS, DHODH, DTYMK, RRM1, RRM2, TK1, TYMS, and UCK2 were unfavorable prognostic markers in patients with lung cancer (Fig. 5a). However, consistent with the decreased expression levels of NR5C2 and TK2 in lung cancer tissues, patients with higher expression levels of NR5C2 and TK2 had better prognosis than patients with low expression levels of those genes (Fig. 5a).

We also showed that contrast with the unfavorable prognosis of pyrimidine metabolic rate–limiting enzymes, purinergic receptors P2RX1, P2RX2, P2RX7, P2RY12, P2RY13, and P2RY14 were favorable prognostic markers in patients with lung cancer (Fig. 5b). However, other purinergic receptors had no prognostic effects.

### Expression levels of pyrimidine metabolic rate–limiting enzymes are associated with the tumor overall survival in lung adenocarcinoma: analysis from The Cancer Genome Atlas lung adenocarcinoma dataset

**Fig. 6:**
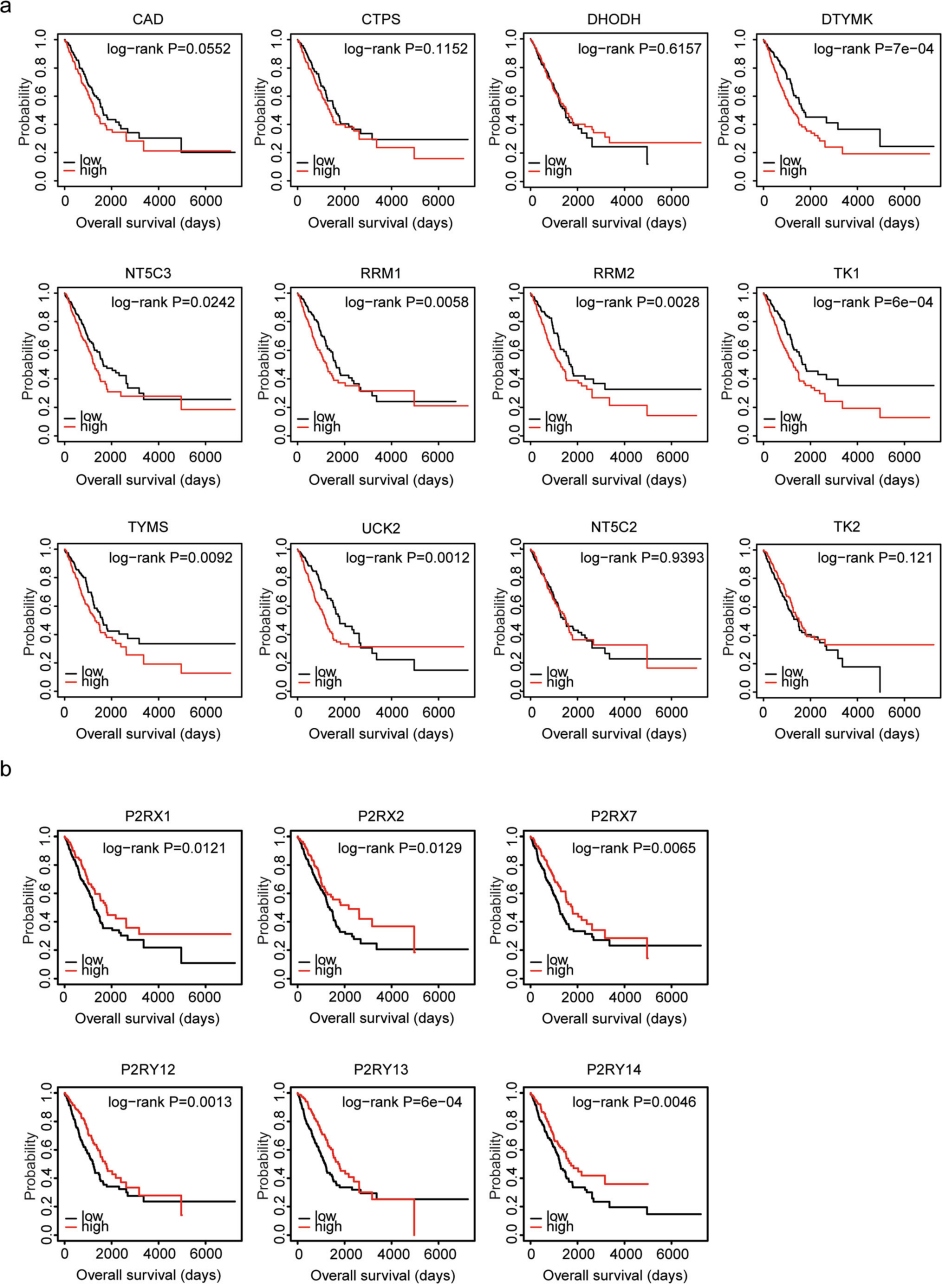
Expression levels of pyrimidine metabolic rate–limiting enzymes are associated with the tumor overall survival in lung adenocarcinoma: analysis from TCGA LUAD dataset. **a** The Kaplan-Meier plotters demonstrated the associations between pyrimidine metabolic rate–limiting enzymes and overall survival in lung cancer using the TCGA LUAD dataset. The log-rank test was used to determine the overall survival *P* value. **b** The Kaplan-Meier plotters demonstrated the prognostic effects of the expression levels of purinergic receptors P2RX1, P2RX2, P2RX7, P2RY12, P2RY13, and P2RY14 in lung cancer

Furthermore, using TCGA LUAD dataset, we confirmed the prognostic effects of pyrimidine metabolic rate–limiting enzymes and purinergic receptors. Similarly, the Kaplan-Meier survival analysis showed that pyrimidine metabolic rate–limiting enzymes DTYMK, NT5C3, RRM1, RRM2, TK1, TYMS, and UCK2 were all associated with adverse prognosis in the lung cancer (Fig. 6a). Patients with high expression levels of DTYMK, NT5C3, RRM1, RRM2, TK1, TYMS, or UCK2 were with low overall survival. However, we found that CAD, CTPS, DHODH, NR5C2, and TK2 had no prognostic effects in TCGA LUAD dataset (Fig. 6a). And only purinergic receptors P2RX1, P2RX2, P2RX7, P2RY12, P2RY13, and P2RY14 were associated with good prognosis in the lung cancer (Fig. 6b).

The prognostic significance of pyrimidine metabolic rate–limiting enzymes in patients with LUSC was also tested using TCGA LUSC dataset. However, unlike LUAD, pyrimidine metabolic rate–limiting enzymes CAD, CTPS, DHODH, DTYMK, NT5C3, RRM1, RRM2, TK1, TYMS, UCK2, and NT5C2 had no prognostic effects in LUSC (Fig. S1). Only high expression levels of TK2 were associated with the adverse prognostic outcomes in LUSC (Fig. S1).

### Increased expression levels of the pyrimidine metabolic rate–limiting enzymes in lung cancer cells are induced by DNA hypomethylation

Next, we tried to determine the mechanisms that induced the high expression levels of pyrimidine metabolic rate–limiting enzymes in lung cancer. The high expression levels of oncogenes are usually mediated by hypo-DNA methylation, DNA amplification, and gene mutation [35]. Using the DNA methylation data deposited in GSE32867 and GSE62948 datasets, we analyzed the DNA methylation intensity of the pyrimidine metabolic rate–limiting enzymes in normal lung tissues and lung cancer tissues.

**Fig. 7:**
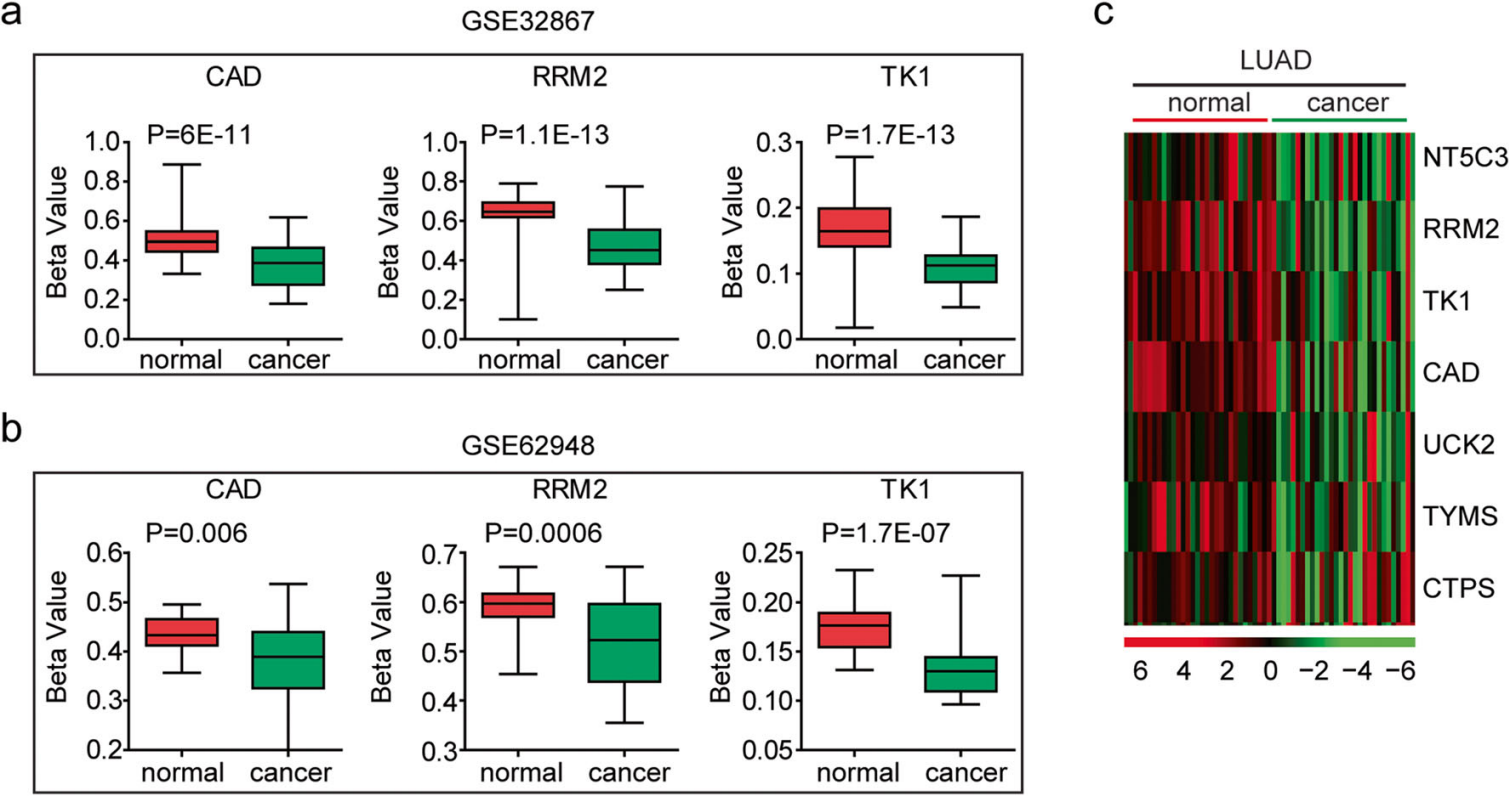
Increased expression levels of the pyrimidine metabolic rate–limiting enzymes in lung cancer cells are induced by DNA hypomethylation. **a** Box plots demonstrated the DNA methylation intensity (*β* value) of pyrimidine metabolic rate limiting enzymes CAD, RRM2, and TK1 in normal lung tissues and lung adenocarcinoma tissues in GSE32867 dataset. **b** Box plots demonstrated the DNA methylation intensity of CAD, RRM2, and TK1 genes in normal lung tissues and lung adenocarcinoma tissues in GSE62948 dataset. **c** Heatmaps demonstrated the methylation level (*β* value) of the pyrimidine metabolic rate–limiting enzymes in normal and tumor tissues in LUAD. Hypermethylated (red), hypomethylated (green) and unchanged (black) genes were delineated

Compared with the lung normal tissues, the pyrimidine metabolic rate–limiting enzymes CAD, RRM2, and TK1 were hypo-methylated in lung cancer tissues derived from GSE32867 dataset (Fig. 7a). Similar results were obtained in GSE62948 dataset that the DNA methylation intensity of CAD, RRM2, and TK1 was lower in lung cancer tissues, compared with normal lung tissues (Fig. 7b). Also, in TCGA LUAD dataset, pyrimidine metabolic rate–limiting enzymes RRM2, TK1, CAD, UCK2, TYMS, and CTPS exhibited hypo-DNA methylation in LUAD tissues (Fig. 7c).

### Increased expression levels of the pyrimidine metabolic rate–limiting enzymes in lung cancer cells are induced by DNA amplification and TP53 mutation

**Fig. 8:**
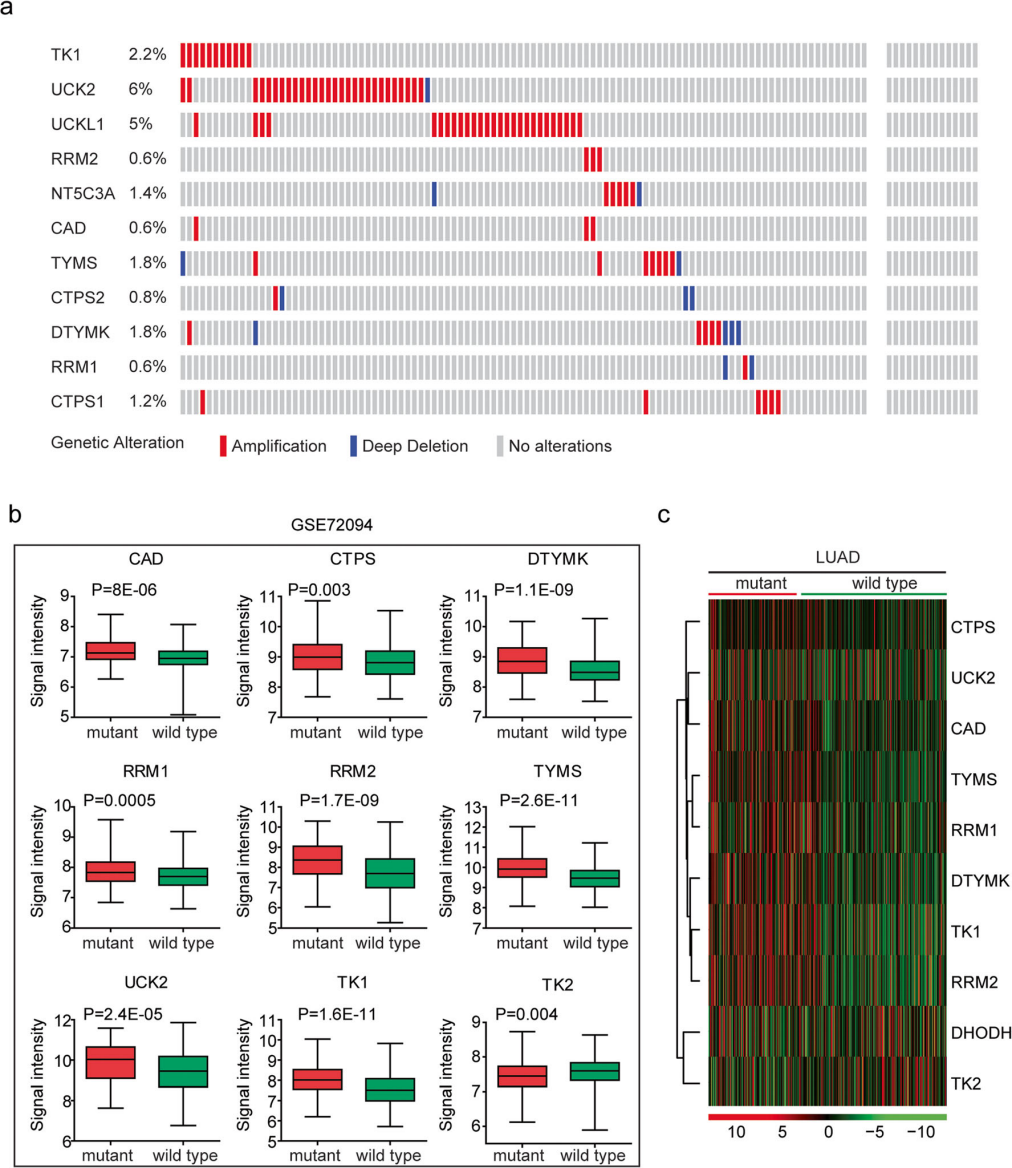
Increased expression levels of the pyrimidine metabolic rate–limiting enzymes in lung cancer cells are induced by DNA amplification and TP53 mutation. **a** Oncoprints demonstrated the alteration frequency of pyrimidine metabolic rate–limiting enzymes in LUAD. Each line represented one patient. **b** Box plots demonstrated the expression levels of the pyrimidine metabolic rate limiting enzymes in patients with lung cancer. *P* values indicated the differences between patients with TP53 mutant and TP53 wild-type. **c** Heatmap demonstrated the expression levels of the pyrimidine metabolic rate–limiting enzymes in TP53 mutant and TP53 wild-type LUAD patients

Another factor determining the activation of pyrimidine metabolic rate–limiting enzymes in lung cancer cells was genomic aberration, particularly DNA amplification. We showed that 6% lung cancer patients were with UCK2 amplification and 5% lung cancer patients were with UCKL1 amplification (Fig. 8a). Also, TK1 amplification occurred in 2.2% lung cancer patients (Fig. 8a). However, other pyrimidine metabolic rate–limiting enzymes were without DNA amplification in lung cancer tissues (Fig. 8a).

TP53 is a critical regulator of multiple metabolism signaling pathways in lung cancer cells [36–38]. Loss of TP53 functions induces uncontrolled pyrimidine synthesis [39]. The present study assessed whether TP53 regulated the expression levels of the pyrimidine metabolic rate–limiting enzymes. We found that pyrimidine metabolic rate–limiting enzymes CAD, CTPS, DTYMK, RRM1, RRM2, TYMS, UCK2, and TK1 were all highly expressed in TP53 mutant lung cancer patients (Fig. 8b). Interestingly, TK2 which was downregulated in lung tumor tissues was highly expressed in lung cancer patients with wild type TP53 (Fig. 8b).

Those results were further validated in the TCGA LUAD dataset. The expression levels of pyrimidine metabolic rate–limiting enzymes CAD, CTPS, DTYMK, RRM1, RRM2, TYMS, UCK2, and TK1 were particularly higher in TP53 mutant lung cancer patients (Fig. 8c). And the expression levels of TK2 were lower in TP53 mutant lung cancer patients (Fig. 8c). Overall, our results suggested that hypo-DNA methylation, DNA amplification, and TP53 mutation were combined contributing to the high expression levels of pyrimidine metabolic rate–limiting enzymes in lung cancer cells.

### Pyrimidine metabolic rate–limiting enzymes are highly correlated in lung cancer

**Fig. 9:**
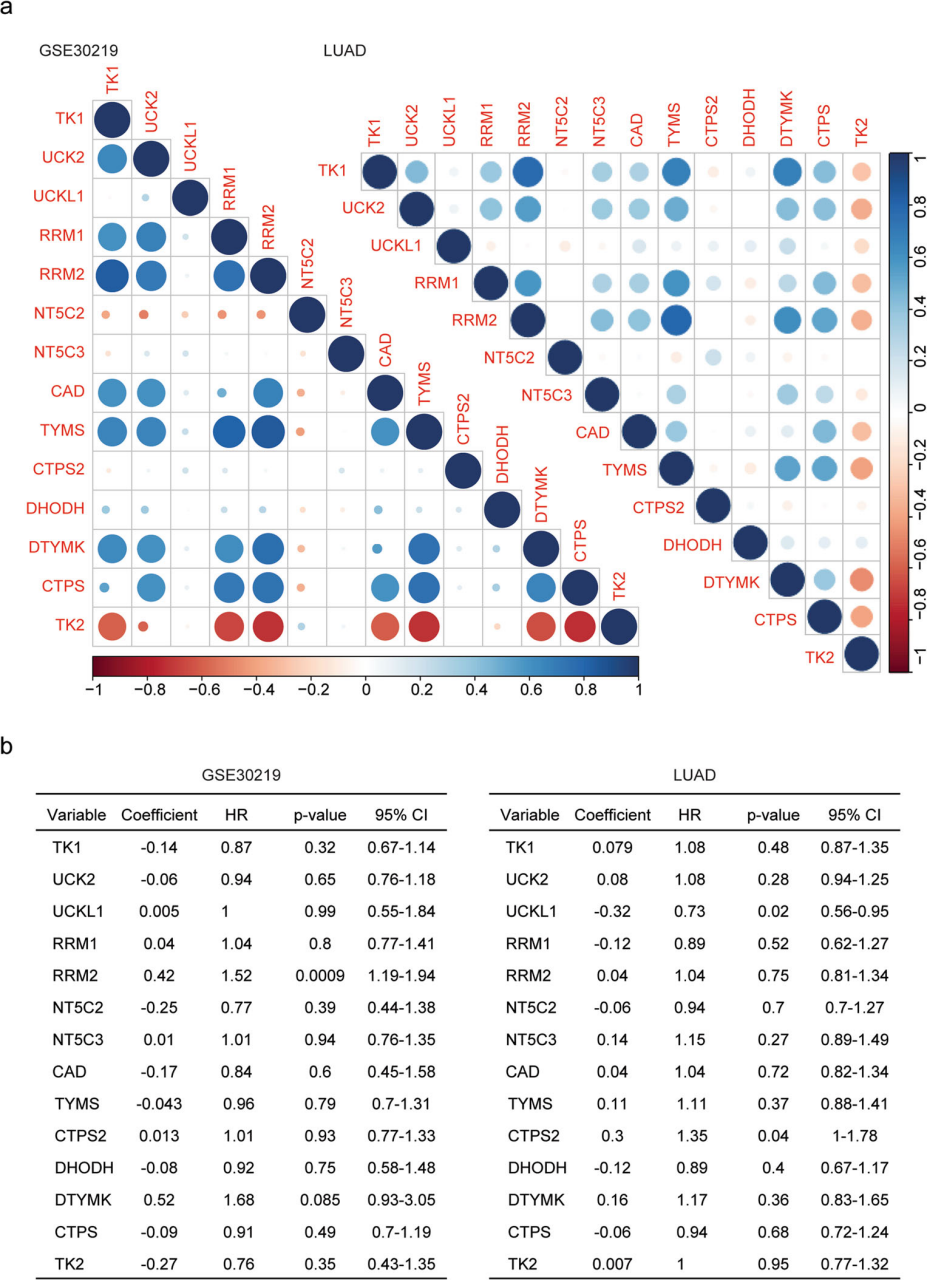
Pyrimidine metabolic rate limiting enzymes are highly correlated in lung cancer. **a** Corrplots demonstrated the correlation between pyrimidine metabolic rate–limiting enzymes in the GSE30219 and TCGA LUAD dataset. The color and the size of the circle represented the correlation coefficients. **b** Multivariate Cox regression was used to test the relationships of pyrimidine metabolic rate–limiting enzyme expressions and overall survival in lung cancer patients in the GSE30219 and TCGA LUAD dataset

Using Spearman’s correlation, we found the high correlations of pyrimidine metabolic rate–limiting enzymes. Particularly, RRM2 was highly associated with TK1, RRM1, TYMS, and DTYMK in GSE30219 dataset (Fig. 9a). However, NT5C2 and TK2 were negatively correlated with other pyrimidine metabolic rate–limiting enzymes (Fig. 9a). Similar results were obtained from TCGA LUAD dataset. RRM2 was positively correlated with other pyrimidine metabolic rate–limiting enzymes, while TK2 was negatively correlated with other pyrimidine metabolic rate–limiting enzymes (Fig. 9a).

Furthermore, we used multivariate Cox regression analysis to determine the connections between the pyrimidine metabolic rate–limiting enzymes. It was revealed that RRM2 was an independent prognostic marker in lung cancer in GSE30219 dataset (Fig. 9b). In LUAD dataset, all pyrimidine metabolic rate–limiting enzymes were interconnected with each other and those genes were not independent prognostic markers (Fig. 9b).

### Combined pyrimidine metabolic rate–limiting enzymes have significant prognostic effects of in lung cancer

**Fig. 10:**
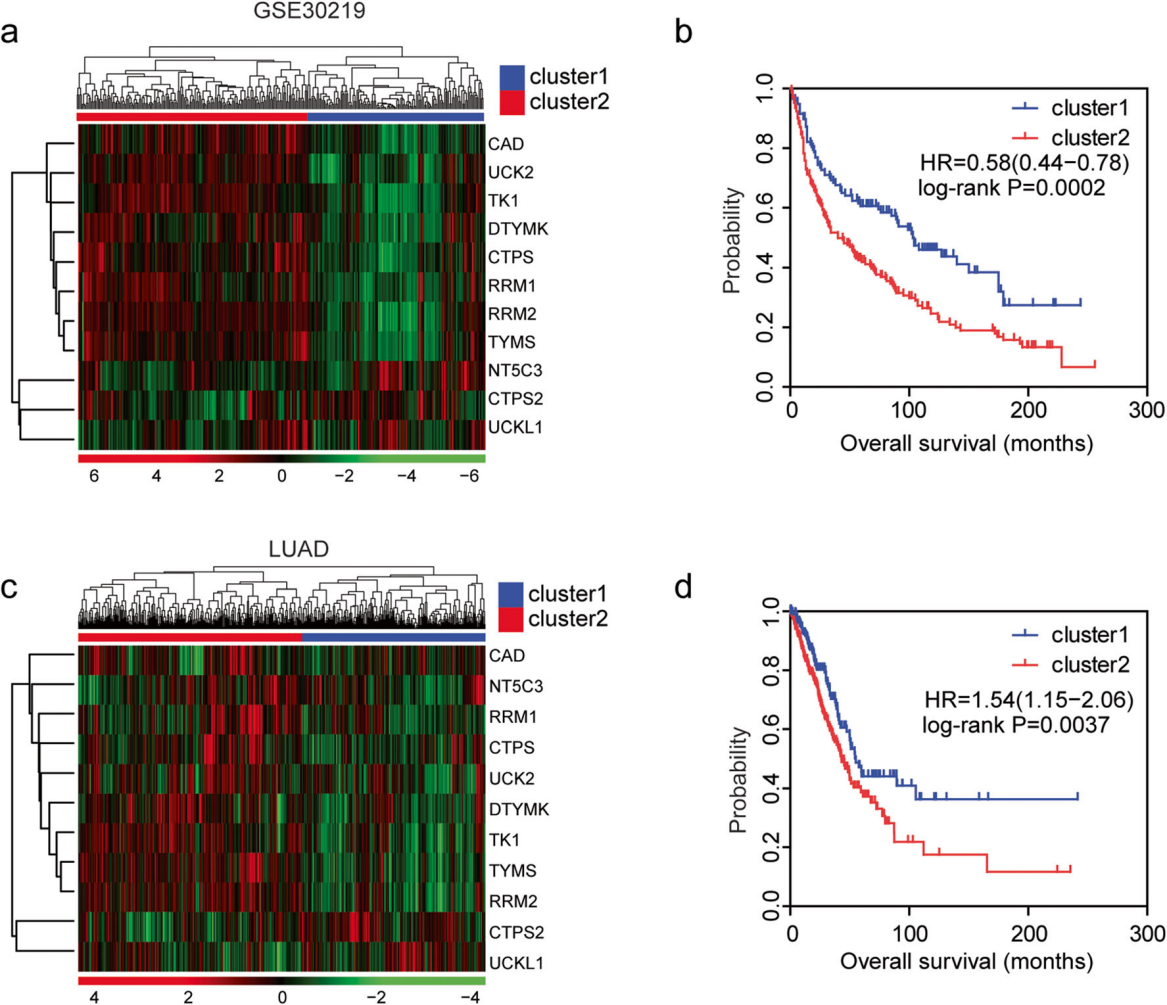
Combined pyrimidine metabolic rate–limiting enzymes have significant prognostic effects of in lung cancer. **a** Unsupervised clustering heatmap showed the division of two clusters of lung cancer patients by the expression levels of pyrimidine metabolic rate–limiting enzymes in GSE30219 dataset. Each line represented one patient. **b** The Kaplan-Meier plotter demonstrated the different clinical outcomes of those two clusters of lung cancer patients in GSE30219 dataset. The log-rank test was used to determine the overall survival *P* value. **c** Unsupervised clustering heatmap showed the division of two clusters of lung cancer patients by the expression levels of pyrimidine metabolic rate–limiting enzymes in TCGA LUAD dataset. **d** The Kaplan-Meier plotter demonstrated the different clinical outcomes of those two clusters of lung cancer patients in TCGA LUAD dataset. The log-rank test was used to determine the overall survival *P* value

Next, we tested the combined prognostic effects of pyrimidine metabolic rate–limiting enzymes in lung cancer. Lung cancer patients were divided into two clusters based on the unsupervised clustering of the expression levels of pyrimidine metabolic rate–limiting enzymes in GSE30219 dataset (Fig. 10a). The cluster1 lung patients were with lower expression levels of CAD, CTPS, RRM1, RRM2, DTYMK, TK1, TYMS, and UCK2 (Fig. 10a). Lung cancer patients in cluster1 were with longer overall survival time, compared with lung cancer patients in cluster 2 (Fig. 10b).

Similarly, the patients were divided into two clusters by the unsupervised clustering of the pyrimidine metabolic rate–limiting enzymes in TCGA LUAD dataset (Fig. 10c). CAD, CTPS, RRM1, RRM2, DTYMK, TK1, TYMS, and UCK2 were downregulated in cluser1 lung cancer patients (Fig. 10c). Lung cancer patients in cluster1 demonstrated better prognostic outcomes, compared with lung cancer patients in cluster 2 (Fig. 10d).

### Pyrimidine metabolic rate–limiting enzymes are upregulated in multiple types of tumor

**Fig. 11:**
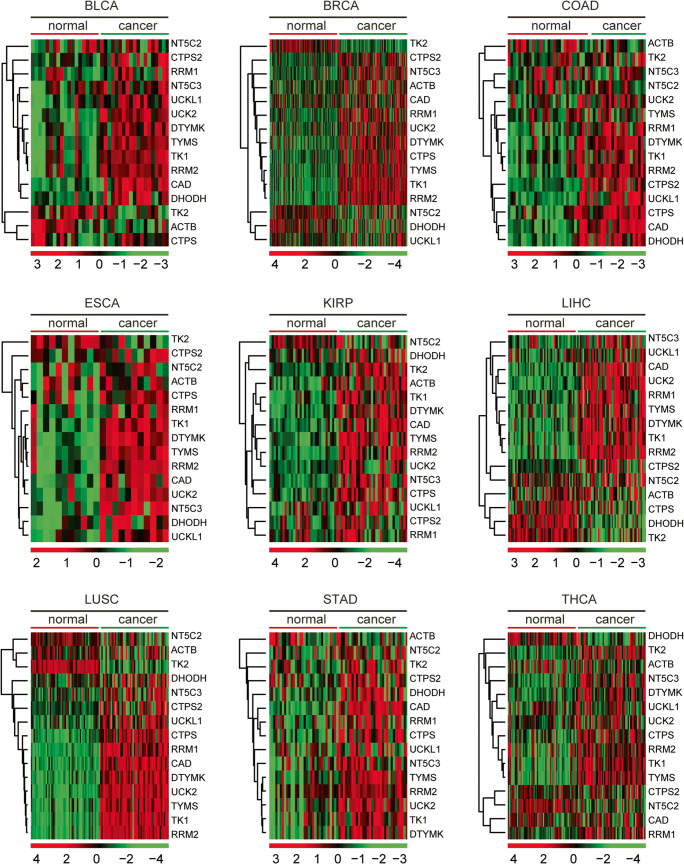
Pyrimidine metabolic rate limiting enzymes are up-regulated in multiple types of tumor. Heatmaps demonstrated the expression levels (log2 count) of pyrimidine metabolic rate limiting enzymes in normal and tumor samples in BLCA, BRCA, COAD, ESCA, KIRP, LIHC, LUSC, STAD, and THCA. Upregulated (red), downregulated (green), and unchanged (black) genes were delineated. BLCA, bladder urothelial carcinoma; BRCA, breast invasive carcinoma; COAD, colon adenocarcinoma; ESCA, esophageal carcinoma; KIRP, kidney renal papillary cell carcinoma; LIHC, liver hepatocellular carcinoma; LUSC, lung squamous cell carcinoma; STAD, stomach adenocarcinoma; THCA, thyroid cancer

Comprehensively, using TCGA database, we investigated the expression levels of pyrimidine metabolic rate–limiting enzymes across different types of cancer. The expression levels of the pyrimidine metabolic rate–limiting enzymes in normal tissues and corresponding tumor tissues were investigated in bladder urothelial carcinoma (BLCA), breast invasive carcinoma (BRCA), colon adenocarcinoma (COAD), esophageal carcinoma (ESCA), kidney renal papillary cell carcinoma (KIRP), liver hepatocellular carcinoma (LIHC), lung squamous cell carcinoma (LUSC), stomach adenocarcinoma (STAD), and thyroid cancer (THCA) (Fig. 11). As illustrated in the heatmaps, pyrimidine metabolic rate–limiting enzymes CAD, CTPS, CTPS2, DHODH, DTYMK, NT5C3, RRM1, RRM2, TK2, TYMS, UCK2, and UCKL1 were highly expressed in tumor tissues (Fig. 11). However, TK2 and NT5C2 were not significantly upregulated in tumor tissues (Fig. 11). Moreover, β-actin was not altered in cancer tissues in most types of tumor (Fig. 11). These results indicated the universal importance of pyrimidine metabolic rate–limiting enzymes in the development of cancer.

Using GSEA assay, we found that pyrimidine metabolism signaling pathway was only significantly enriched in BRCA and THCA (Fig. S2). Although, pyrimidine metabolic rate–limiting enzymes were upregulated in BLCA, COAD, ESCA, LIHC, and STAD, the pyrimidine metabolism signaling pathway was not significantly enriched (Fig. S2).

### The association between the expression levels of pyrimidine metabolic rate–limiting enzymes and the tumor overall survival in liver cancer, breast cancer, or stomach cancer: analysis from breast invasive carcinoma, stomach adenocarcinoma, and liver hepatocellular carcinoma datasets

Like LUAD, pyrimidine metabolic rate–limiting enzymes were highly expressed in BRCA, LIHC, and STAD. However, in TCGA BRCA dataset, pyrimidine metabolic rate–limiting enzymes CAD, CTPS, DHODH, DTYMK, NT5C3, RRM1, RRM2, TK1, TYMS, UCK2, NT5C2, or TK2 demonstrated no prognostic effect (Fig. S3). Similarly, expression levels of pyrimidine metabolic rate–limiting enzymes had no clinical relevance in stomach cancer (Fig. S4). Only, TK1 was associated with better clinical outcomes (Fig. S4).

**Fig. 12:**
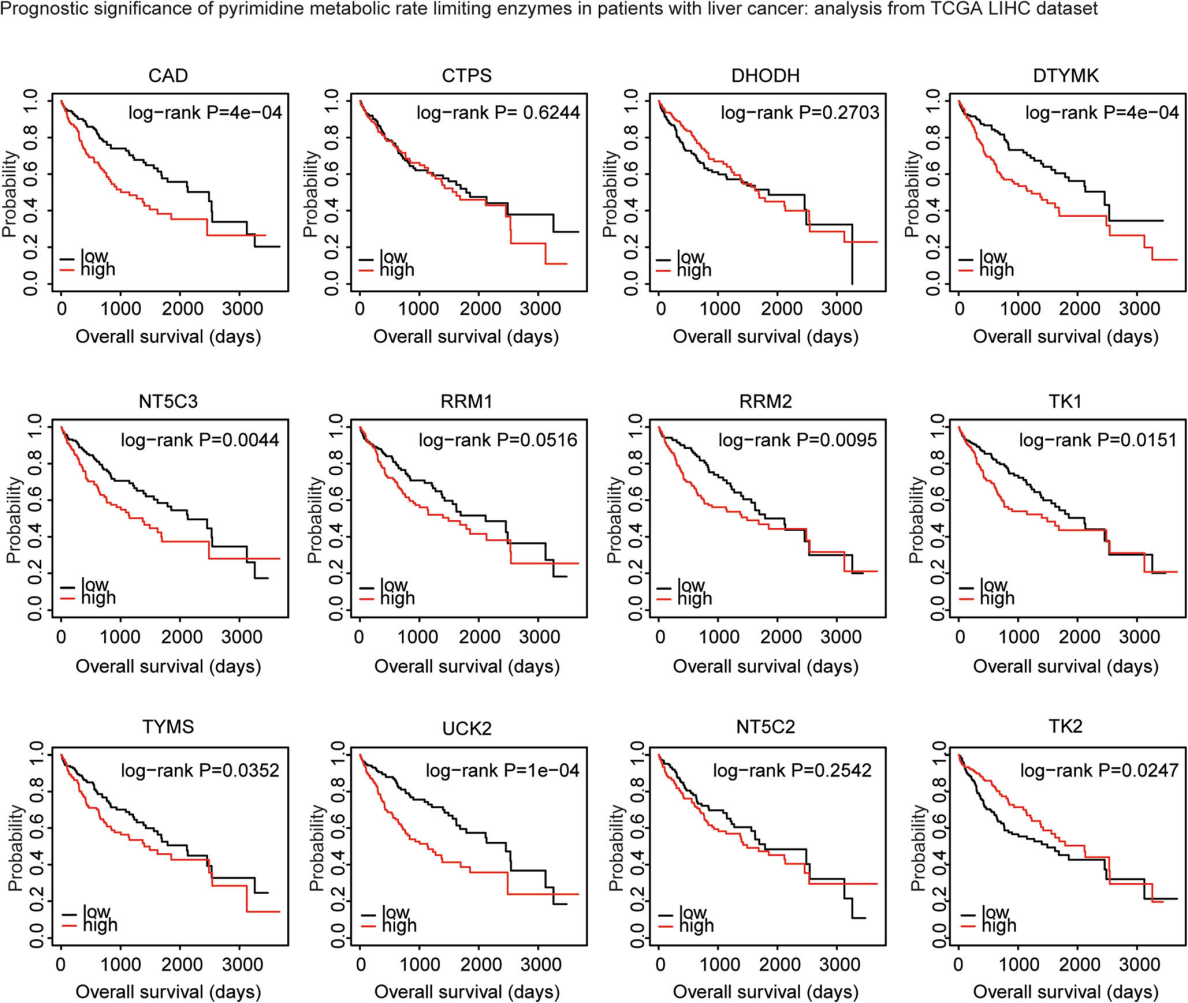
Expression levels of pyrimidine metabolic rate–limiting enzymes are associated with the overall survival in liver cancer: analysis from TCGA LIHC dataset. The Kaplan-Meier plotters demonstrated the associations between pyrimidine metabolic rate limiting enzymes and overall survival in liver cancer using the TCGA LIHC dataset. The log-rank test was used to determine the overall survival *P* value. LIHC: liver hepatocellular carcinoma

On the contrary, high expression levels of pyrimidine metabolic rate–limiting enzymes CAD, DTYMK, NT5C3, RRM1, RRM2, TK1, TYMS, and UCK2 were all associated with worse clinical outcomes in TCGA LIHC dataset (Fig. 12). Moreover, patients with higher expression levels of TK2 had better prognosis than patients with low expression levels of TK2 (Fig. 12). Those results highlighted the different prognostic effects of pyrimidine metabolic rate–limiting enzymes in different tumor types.

## Discussion

Metabolic reprogramming is a hallmark of cancer [20] and provides critical information for cancer classification and clinical prognosis [40]. Here, we identified the clinical relevance of pyrimidine metabolic rate–limiting enzymes in lung cancer based on their mRNA expression patterns using GEO and TCGA datasets. Ideally, we should use the metabolic activities to determine the prognostic effects of the pyrimidine metabolic rate–limiting enzymes. However, previous results suggested that the metabolic reprogramming was caused by gene expression changes [41] and the expression profiles of metabolic pathway genes reflected the actual metabolic activities [42]. So, the expression patterns of metabolic genes had potential implications for clinical prognosis.

Lung cancer is a heterogeneous disease, including many different subtypes with different genetic and epigenetic abnormality [6]. Because of the complexity of lung cancer [43], results derived from GEO and TCGA datasets were not always consistent with each other. Also, the difference in treatment protocol and microarray platform in individual study limited the further applications of these findings [44]. For example, CAD, CTPS, and DHODH had no prognostic effect in LUAD dataset but associated with the clinical outcomes in lung cancer patients derived from GEO datasets. To address this problem, we collected and studied multiple lung cancer GEO datasets and TCGA datasets. Our results suggested that the converged pyrimidine metabolism signaling pathway was generally altered in many datasets. And the pyrimidine metabolic rate–limiting enzymes DTYMK, NT5C3, RRM1, RRM2, TK1, TYMS, and UCK2 had particular values in lung cancer prognosis. Those highly expressed pyrimidine metabolic rate–limiting enzyme increased pyrimidine metabolism, facilitated the uncontrolled cell proliferation, and changed of the immune cell responses.

Some of the pyrimidine metabolic rate–limiting enzymes, such as TK1 [45], UCK2 [46], and RRM1 [47], were reported to be associated with the poor outcomes of lung cancer in systematic review or meta-analysis. Functional studies identified pyrimidine metabolic rate–limiting enzymes DHODH [48] and DTYMK [49] as therapeutic targets in lung cancer. Using the GEO and TCGA datasets, we confirmed the prognostic significance of pyrimidine metabolic rate–limiting enzymes TK1, UCK2, and RRM1 in LUAD. Furthermore, we found that pyrimidine metabolic rate–limiting enzymes CAD, RRM2, DTYMK, TYMS, TK2, and NR5C2 were all associated with the clinical outcomes of lung cancer and liver cancer. However, the expression levels and prognostic effects of purinergic receptors in lung cancer were complex. It was reported that P2RX7 increased cancer invasiveness and metastasis and was adverse prognostic factor [50, 51]. However, in our data, we found that P2RX7 was downregulated and was a good prognostic factor in lung cancer. Moreover, purinergic receptors P2RX1, P2RY12, P2RY13, and P2RY14 shared similar expression profiling and prognostic relevance. Those results suggested the complex functions of purinergic receptors in cancer development and should be further studied. Particularly, the purinergic receptors may influence the tumor immune cell responses by altering the tumor microenvironment.

The present study provided potential biomarkers for clinical prognosis of lung cancer. However, there were some limitations in this study. First, although the expression profiles of metabolic genes reflected the actual metabolic activities, the enzymatic activities of pyrimidine metabolic rate–limiting enzymes should be further tested. Second, clinical validations and functional studies were needed to reveal the inner mechanisms of how pyrimidine metabolic rate–limiting enzymes correlated with the clinical outcomes of lung cancer patients. Our results also suggested the different prognostic effects of pyrimidine metabolic rate–limiting enzymes in LUAD and LUSC. So, the clinical relevance the pyrimidine metabolic rate–limiting enzymes in different subtypes of lung cancer should also be further illustrated. In our further studies, we will address those limitations and provide a more precise and reliable prognostic signature based on the metabolic activities of the pyrimidine metabolic rate–limiting enzymes.

## Electronic supplementary material

Fig. S1. The Kaplan-Meier Plotters demonstrated the associations between pyrimidine metabolic rate limiting enzymes and overall survival in lung squamous cell carcinoma using the TCGA LUSC dataset. The log-rank test was used to determine the overall survival *P*-value. LUSC: lung squamous cell carcinoma (TIF 1308 kb)

High resolution image (PNG 2798 kb)

Fig. S2. Enrichment plots demonstrated the enriched pyrimidine metabolism signaling pathway in BLCA, BRCA, COAD, ESCA, HNSC, LIHC, LUSC, STAD and THCA datasets. Enrichment of NES and *P*-values were presented. BLCA: bladder urothelial carcinoma; BRCA: breast invasive carcinoma; COAD: colon adenocarcinoma; ESCA: esophageal carcinoma; HNSC: head and neck cancer; LIHC: liver hepatocellular carcinoma; LUSC: lung squamous cell carcinoma; STAD, stomach adenocarcinoma; THCA: thyroid cancer (TIF 1128 kb)

High resolution image (PNG 1167 kb)

Fig. S3. The Kaplan-Meier Plotters demonstrated the associations between pyrimidine metabolic rate limiting enzymes and overall survival in breast cancer using the TCGA BRCA dataset. The log-rank test was used to determine the overall survival P-value. BRCA: breast invasive carcinoma (TIF 1289 kb)

High resolution image (PNG 2733 kb)

Fig. S4. The Kaplan-Meier Plotters demonstrated the associations between pyrimidine metabolic rate limiting enzymes and overall survival in stomach cancer using the TCGA STAD dataset. The log-rank test was used to determine the overall survival P-value. STAD, stomach adenocarcinoma (TIF 1303 kb)

High resolution image (PNG 2822 kb)

## Data Availability

The datasets generated and/or analyzed during the current study are available in TCGA (tcga.xenahubs.net) and GEO (www.ncbi.nlm.nih.gov/geo) repositories.
