# Supplementary figures and images for: High expression levels of pyrimidine metabolic rate–limiting enzymes are adverse prognostic factors in lung adenocarcinoma: a study based on The Cancer Genome Atlas and Gene Expression Omnibus datasets
Source: Purinergic Signal. 2020 Jul 8;16(3):347–66. doi: 10.1007/s11302-020-09711-4 (PMC7524999; doi:10.1007/s11302-020-09711-4)

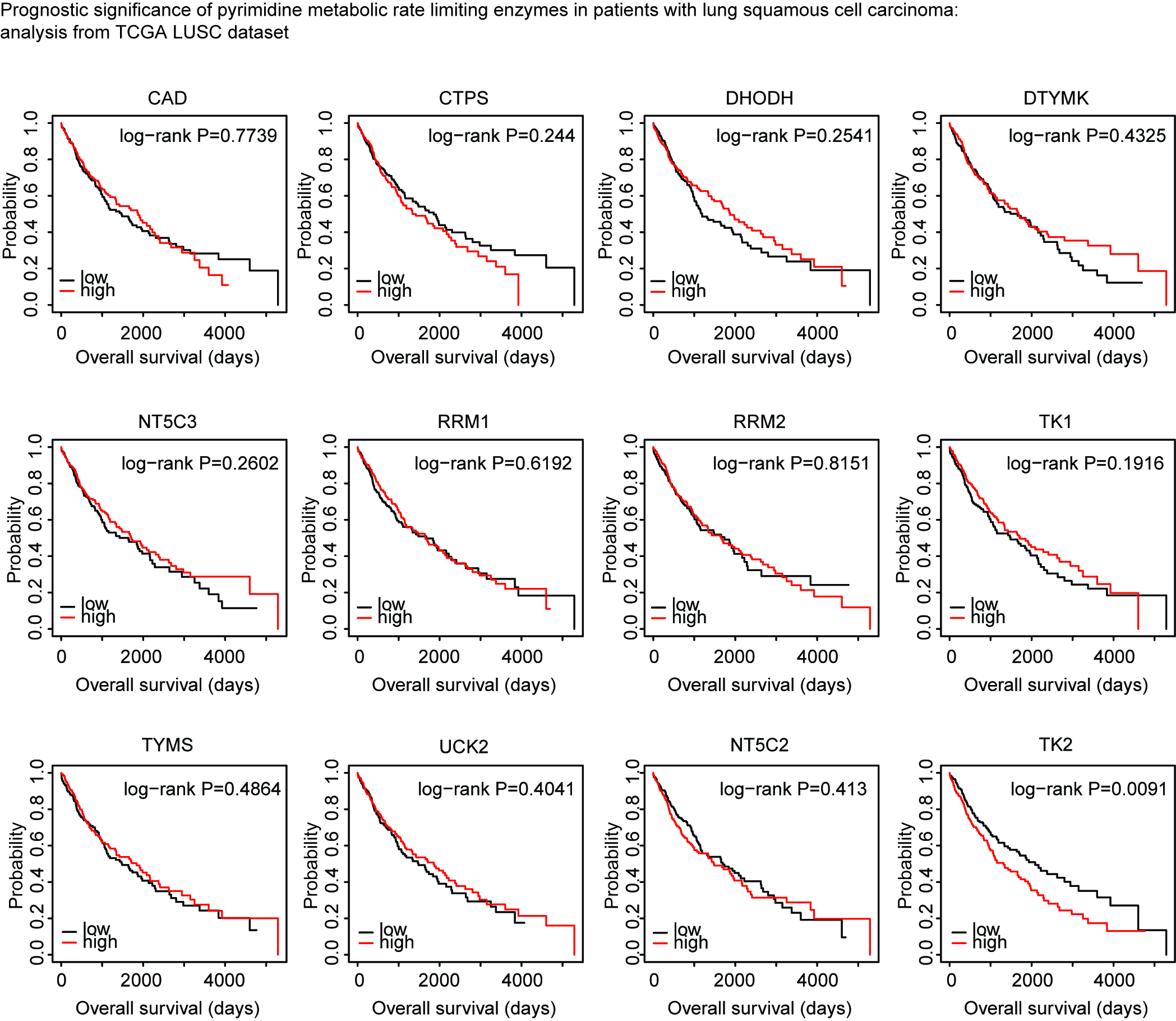

Supplement: Supplementary file 1 — The Kaplan-Meier Plotters demonstrated the associations between pyrimidine metabolic rate limiting enzymes and overall survival in lung squamous cell carcinoma using the TCGA LUSC dataset. The log-rank test was used to determine the overall survival P-value. LUSC: lung squamous cell carcinoma (TIF 1308 kb) [file 11302_2020_9711_MOESM1_ESM.tif]

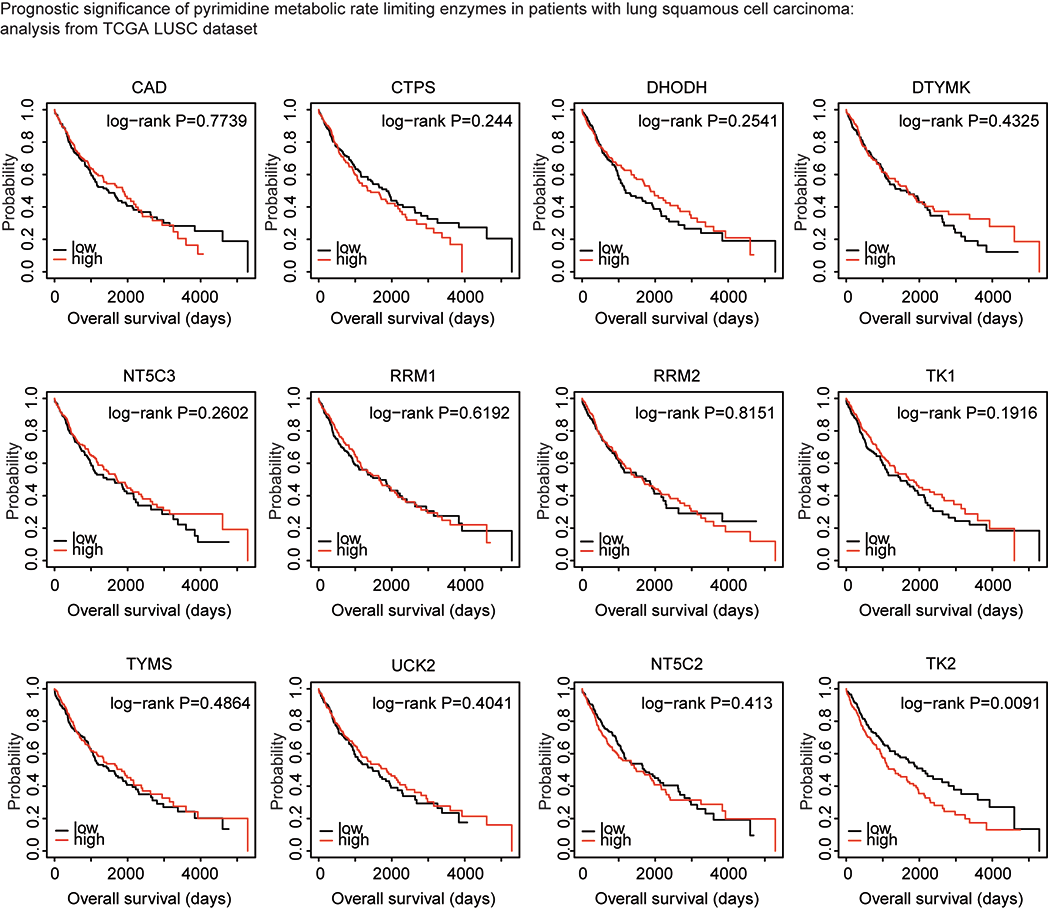

Supplement: Supplementary file 2 — High resolution image (PNG 2798 kb) [file 11302_2020_9711_Fig13_ESM.png]

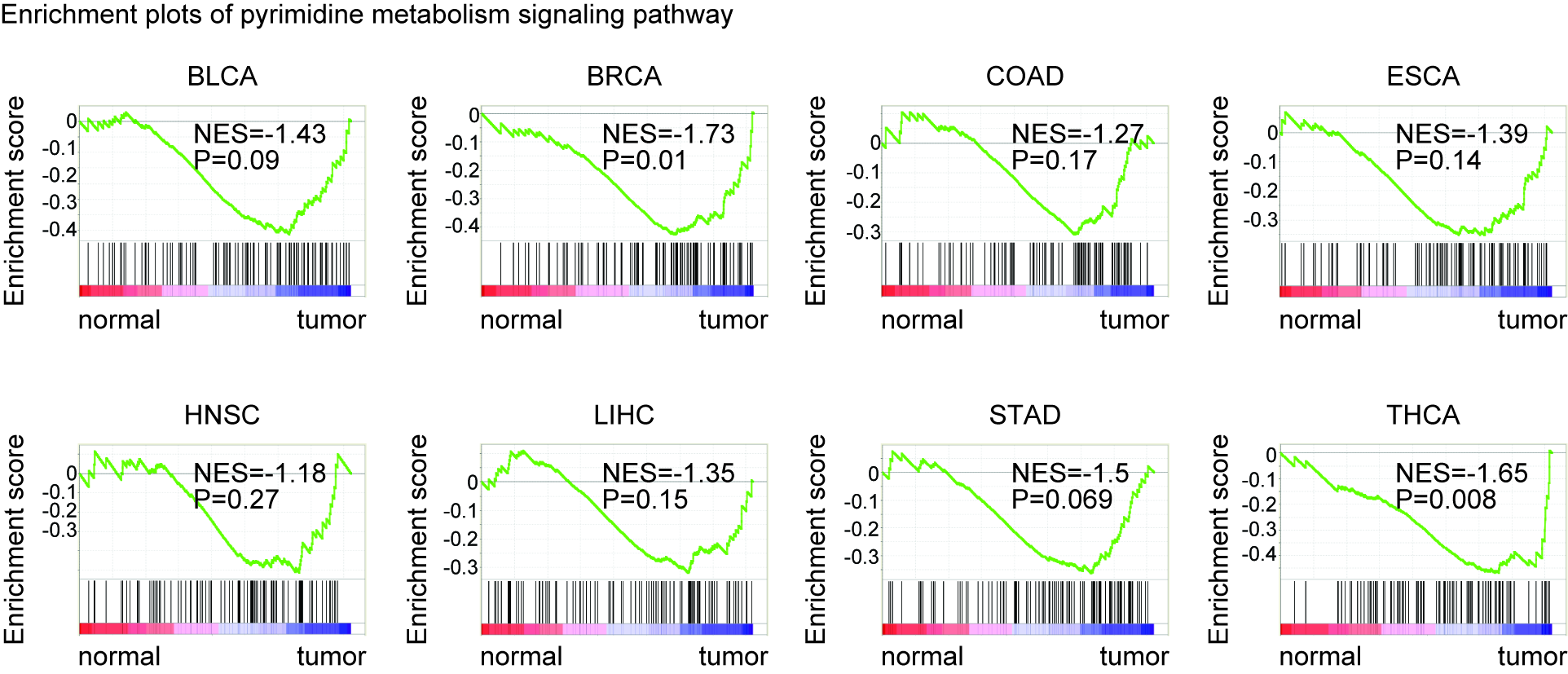

Supplement: Supplementary file 3 — Enrichment plots demonstrated the enriched pyrimidine metabolism signaling pathway in BLCA, BRCA, COAD, ESCA, HNSC, LIHC, LUSC, STAD and THCA datasets. Enrichment of NES and P-values were presented. BLCA: bladder urothelial carcinoma; BRCA: breast invasive carcinoma; COAD: colon adenocarcinoma; ESCA: esophageal carcinoma; HNSC: head and neck cancer; LIHC: liver hepatocellular carcinoma; LUSC: lung squamous cell carcinoma; STAD, stomach adenocarcinoma; THCA: thyroid cancer (TIF 1128 kb) [file 11302_2020_9711_MOESM2_ESM.tif]

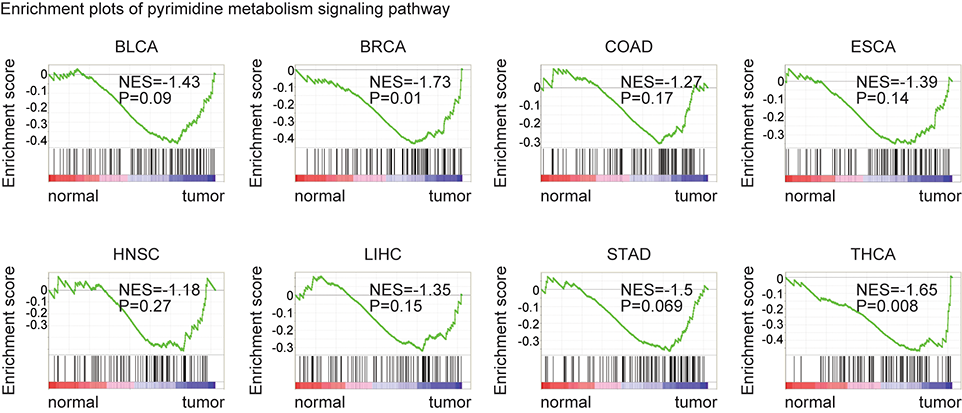

Supplement: Supplementary file 4 — High resolution image (PNG 1167 kb) [file 11302_2020_9711_Fig14_ESM.png]

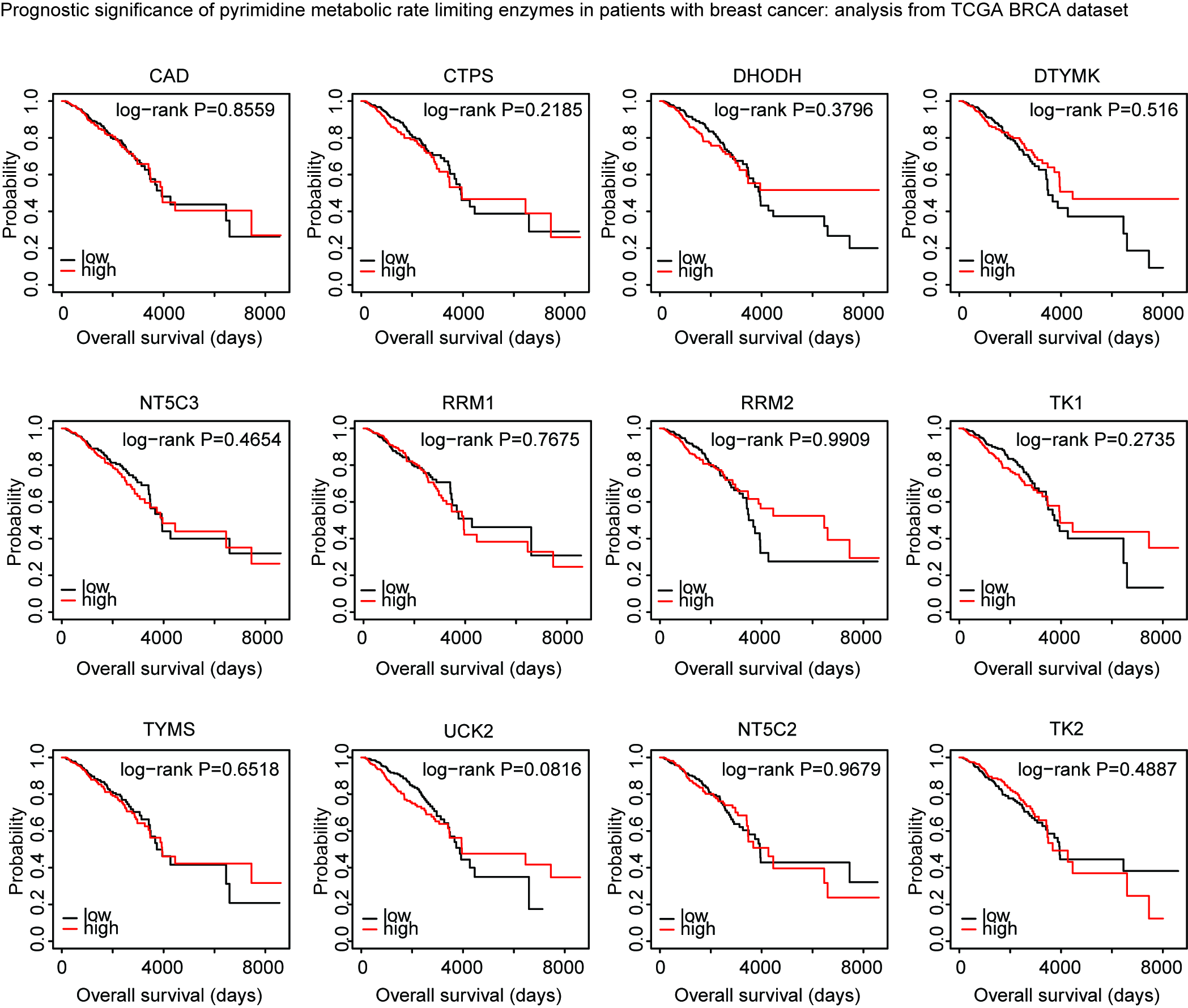

Supplement: Supplementary file 5 — The Kaplan-Meier Plotters demonstrated the associations between pyrimidine metabolic rate limiting enzymes and overall survival in breast cancer using the TCGA BRCA dataset. The log-rank test was used to determine the overall survival P-value. BRCA: breast invasive carcinoma (TIF 1289 kb) [file 11302_2020_9711_MOESM3_ESM.tif]

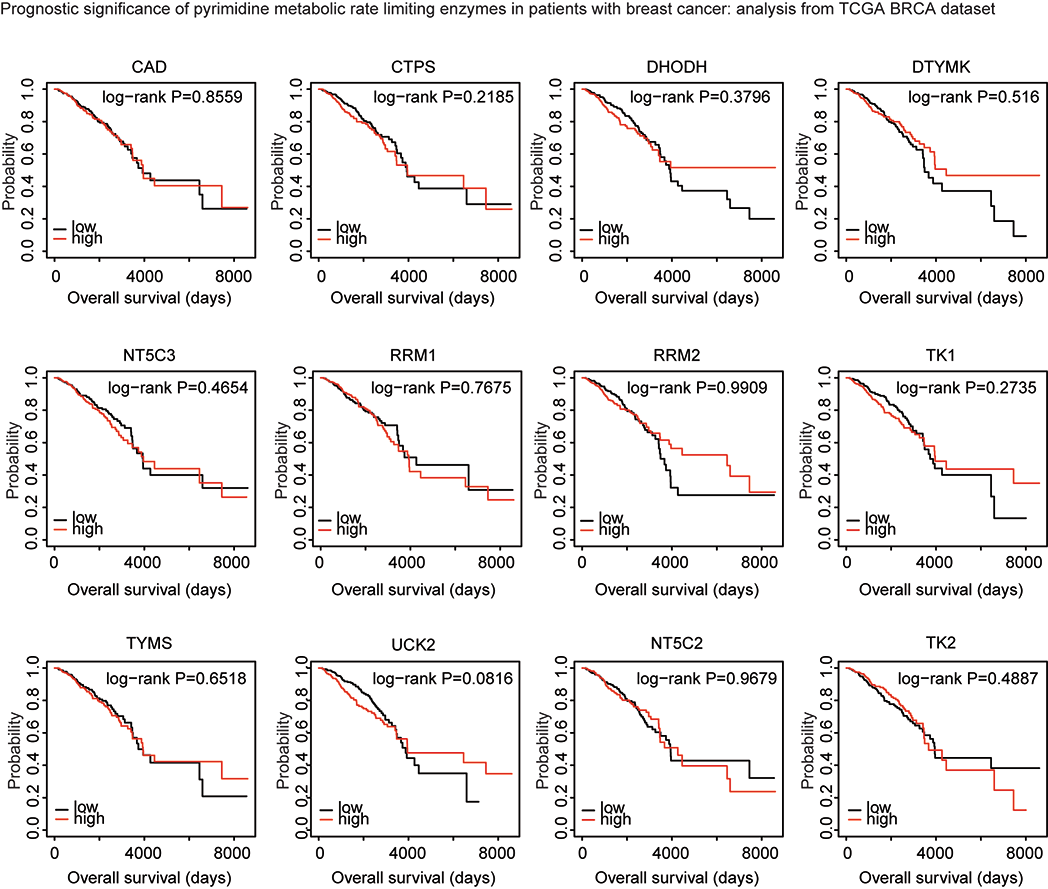

Supplement: Supplementary file 6 — High resolution image (PNG 2733 kb) [file 11302_2020_9711_Fig15_ESM.png]

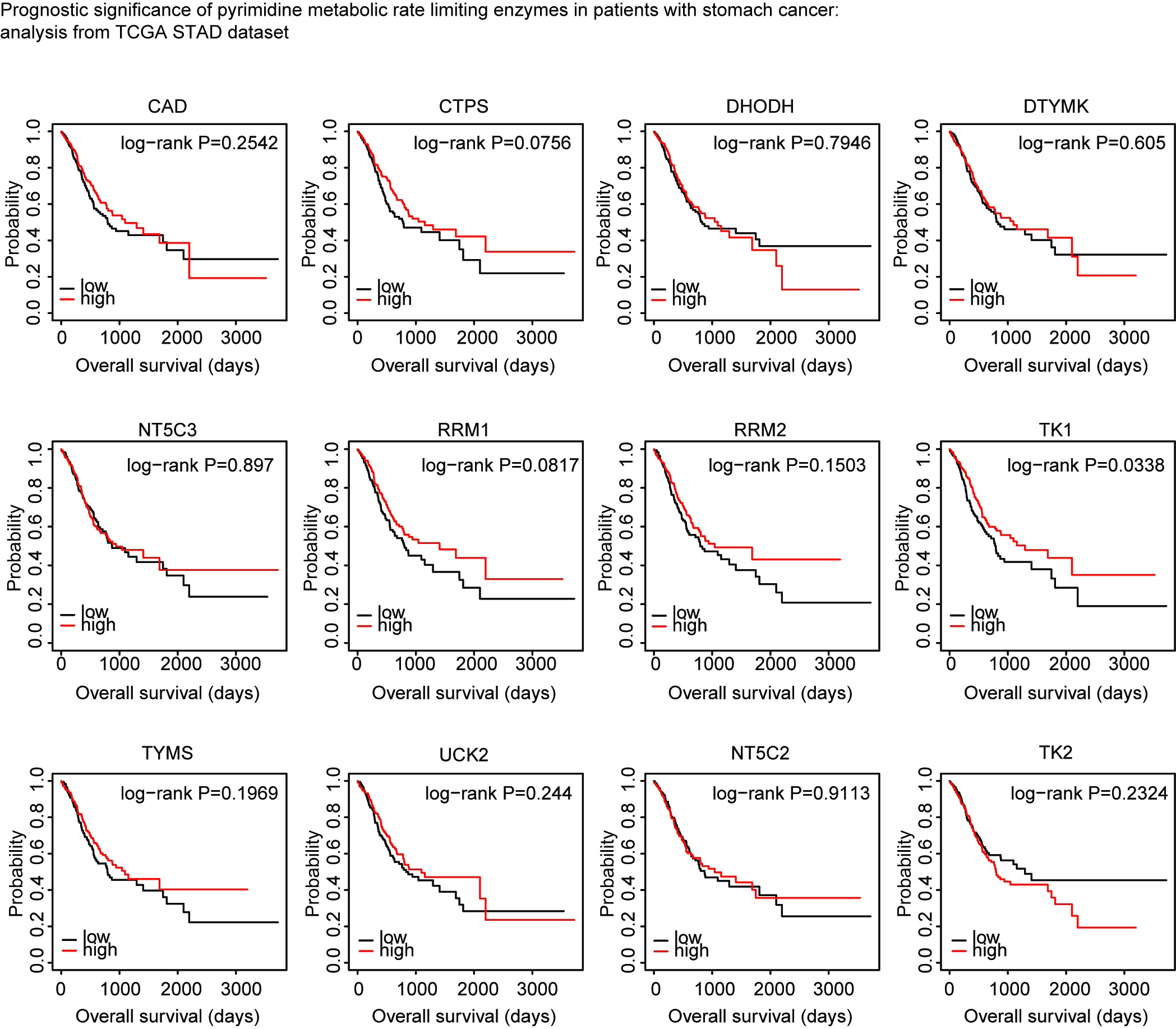

Supplement: Supplementary file 7 — The Kaplan-Meier Plotters demonstrated the associations between pyrimidine metabolic rate limiting enzymes and overall survival in stomach cancer using the TCGA STAD dataset. The log-rank test was used to determine the overall survival P-value. STAD, stomach adenocarcinoma (TIF 1303 kb) [file 11302_2020_9711_MOESM4_ESM.tif]

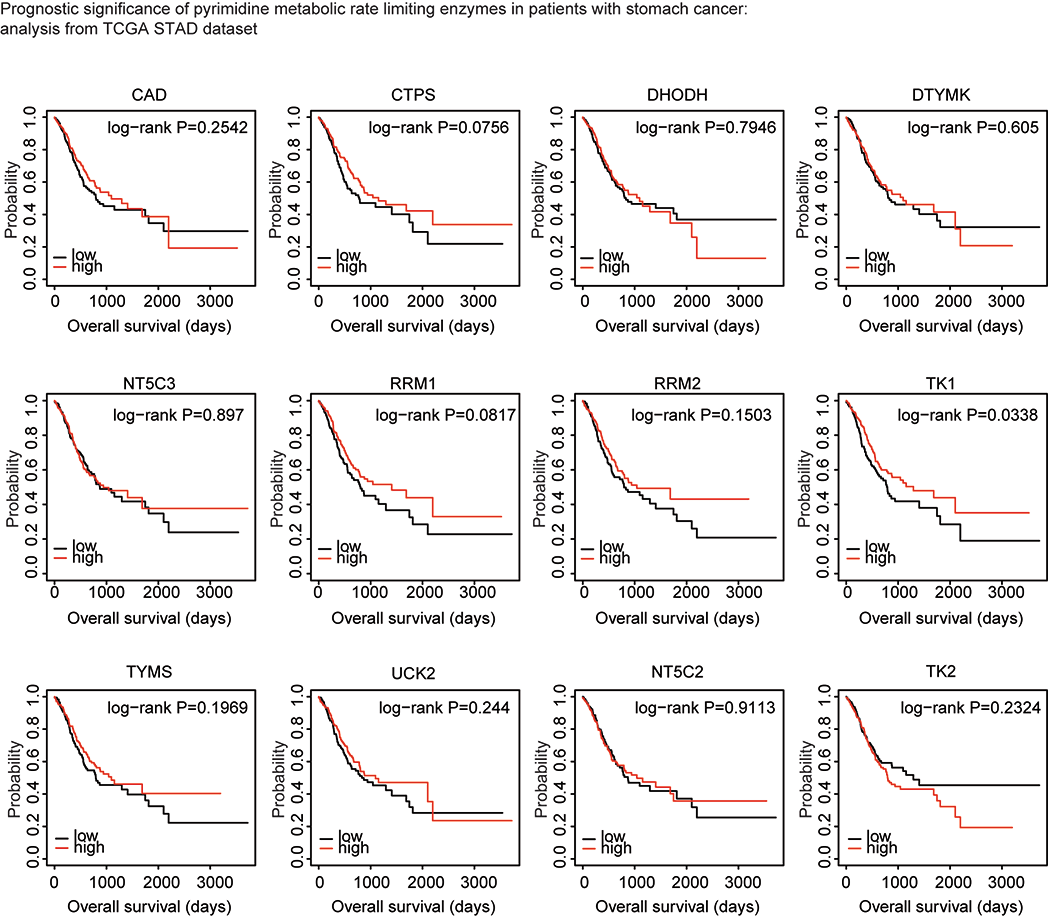

Supplement: Supplementary file 8 — High resolution image (PNG 2822 kb) [file 11302_2020_9711_Fig16_ESM.png]
